# A fast iris recognition system through optimum feature extraction

**DOI:** 10.7717/peerj-cs.184

**Published:** 2019-04-08

**Authors:** Humayan Kabir Rana, Md. Shafiul Azam, Mst. Rashida Akhtar, Julian M.W. Quinn, Mohammad Ali Moni

**Affiliations:** 1 Department of Computer Science and Engineering, Green University of Bangladesh, Dhaka, Bangladesh; 2 Department of Computer Science and Engineering, Pabna University of Science and Technology, Pabna, Bangladesh; 3 Department of Computer Science and Engineering, Varendra University, Rajshahi, Bangladesh; 4 Bone Biology Division, Garvan Institute of Medical Research, NSW, Australia; 5 School of Medical Sciences, Faculty of Medicine and Health, The University of Sydney, Sydney, Australia

**Keywords:** Biometrics, Iris Recognition, PCA, DWT, Gabor filter, Hough Transformation, Daugman’s Rubber Sheet Model

## Abstract

With an increasing demand for stringent security systems, automated identification of individuals based on biometric methods has been a major focus of research and development over the last decade. Biometric recognition analyses unique physiological traits or behavioral characteristics, such as an iris, face, retina, voice, fingerprint, hand geometry, keystrokes or gait. The iris has a complex and unique structure that remains stable over a person’s lifetime, features that have led to its increasing interest in its use for biometric recognition. In this study, we proposed a technique incorporating Principal Component Analysis (PCA) based on Discrete Wavelet Transformation (DWT) for the extraction of the optimum features of an iris and reducing the runtime needed for iris template classification. The idea of using DWT behind PCA is to reduce the resolution of the iris template. DWT converts an iris image into four frequency sub-bands. One frequency sub-band instead of four has been used for further feature extraction by using PCA. Our experimental evaluation demonstrates the efficient performance of the proposed technique.

## Introduction

Biometric recognition refers to the study of identifying persons based on their unique physical traits or behavioral characteristics (Umer, Dhara & Chanda, 2017). Physical characteristics commonly include an iris, face, fingerprint, retina, vein, voice or hand geometry, while behavioral characteristics may include handwriting, walking gait, signature, and typing keystrokes. To be useful, a biometric requires features that can be accurately analysed to provide unique, and stable information about a person that can be used reliably in authentication applications and many advances have been made in this area (Naseem et al., 2017). The iris has easily accessible and unique features that are stable over the lifetime of an individual. For this reason, iris recognition technology has been widely studied in the field of information security. Iris recognition systems can already be applied to identify individuals in controlled access and security zones, and could feasibly be used for verification of passengers at immigration, airports, stations, computer access at research organization, database access control in distributed systems etc. (Galdi, Nappi & Dugelay, 2016). Iris recognition systems can also be applied in the field of financial services such as banking services and credit card use, and such a system would not have the same vulnerabilities as passwords and numbers. Iris recognition systems are being trialled in many countries for national ID cards, immigration, national border control, airline crews, airport staffs, and missing children identification etc. (Galdi, Nappi & Dugelay, 2016). While there are still some concerns about using iris-based recognition in mass consumer applications due to iris data capturing issues, it is widely believed that, in time, the technology is likely to find its way into common use (Nabti & Bouridane, 2008).

**Figure 1:**
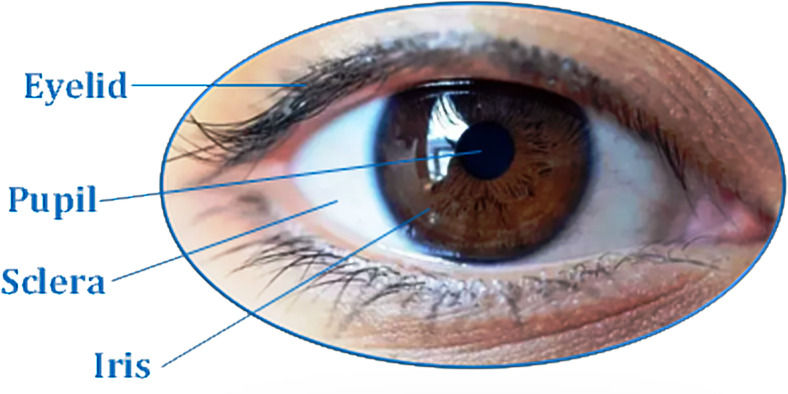
The outer look of a human iris. The iris is a circular structure bounded by the sclera and pupil that controls the amount of light reaching the retina.

An iris is a round contractile membrane of the eye, between sclera and pupil. It begins to form during embryo gestation, being fully formed at around eight months. The uniqueness of the iris patterns comes from the richness of the texture details arising from the crypts, radial furrows, filaments, pigment frills, flecks, stripes and arching ligaments. These give rise to complex and irregular textures that are so randomly distributed as to make the human iris one of the most reliable biometric characteristics. The iris is bounded by the pupil at the inner boundary and the sclera at its outer boundary. These inner and outer boundaries are circular in shape and easily accessible but can be partially blocked by the upper and lower eyelids and eyelashes (Galdi, Nappi & Dugelay, 2016). Figure 1 shows a view of a typical human iris.

In this paper, feature extraction techniques are the main focus. Indeed, the selection of optimal feature subsets has become a crucial issue in the field of iris recognition. To improve feature extraction, we propose that an approach combining Principal Component Analysis (PCA) and Discrete Wavelet Transformation (DWT) will extract the key features of an iris and thereby reduce image resolution and in turn the runtime of the classification or analysis that is required for the resulting iris templates.

## Methodology

**Figure 2:**
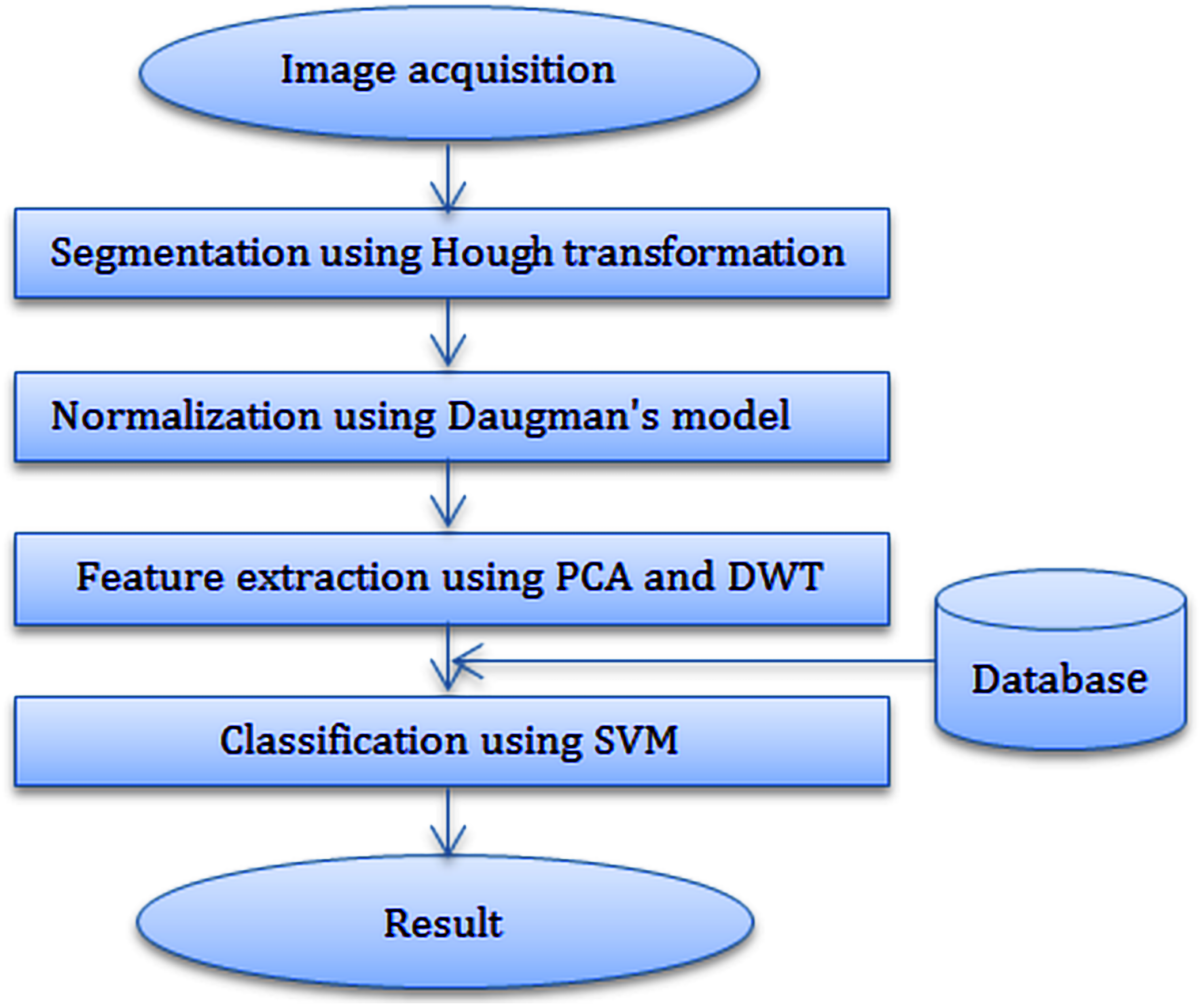
Diagram of iris recognition system. Here, Hough transform, Daugman’s rubber-sheet model, PCA + DWT and SVM are used for segmentation, normalization, feature extraction and classification respectively.

Iris recognition processing generally consists of the following steps: (i) Image acquisition (ii) Iris segmentation (iii) Normalization (iv) Feature extraction and (v) Classification. In our approach presented here, segmentation was achieved using the Hough transform for localizing the iris and pupil regions. The segmented iris region was normalized to a rectangular block with fixed polar dimensions using Daugman’s rubber sheet model. A combined PCA and DWT were applied on a fixed size normalized iris for feature extraction. The Support Vector Machine was used for classification the similarity between the iris templates. Figure 2 shows the system processes that we used for iris recognition.

### Image acquisition

The first step in iris recognition is image acquisition, i.e., the capture of a sequence of high-quality iris images from the subject. These images should clearly show the entire eye, but particularly the iris and pupil. Preprocessing operations may be applied to the captured images in order to enhance their quality and provide sufficient resolution and sharpness (Frucci et al., 2016). In this work, the CASIA-Iris V4 database has been used instead of actual captured eye images (Tieniu Tan, 2015). CASIA-IrisV4 is an extension of CASIA-IrisV3, and contains six subsets that include three subsets (from CASIA-IrisV3), namely CASIA-Iris-Interval, CASIA-Iris-Lamp, and CASIA-Iris-Twins. The three new subsets added to CASIA-IrisV4 are CASIA-Iris-Distance, CASIA-Iris-Syn and CASIA-Iris-Thousand respectively. Center for Biometrics and Security Research (CBSR) captured images of CASIA-Iris-Interval using their self-developed iris camera. Iris images of CASIA-Iris-Interval is good for studying the texture features of iris. Iris images of CASIA-Iris-Lamp were captured with a hand-held iris sensor, and these images are well-suited for finding problems of non-linear iris normalization and robust feature representation. CBSR collected iris images of CASIA-Iris-Twins during Annual Twins Festival in Beijing. CASIA-Iris-Twins data-sets are suitable for studying the similarities and dissimilarities between iris images of twins. CASIA-Iris-Distance images were captured by employing long-range multi-modal biometric image acquisition and recognition system. CASIA-Iris-Thousand database contains 20,000 iris images, which were captured using IKEMB-100 camera. CASIA-Iris-Thousand images are suitable for experimenting the uniqueness of iris features and developing novel iris classification methods. CASIA-IrisV4 contains a total 54,601 iris images. This includes samples from more than 1,800 genuine subjects and 1,000 virtual subjects. All iris images are 8-bit grey-level image and the file format is JPEG (Tieniu Tan, 2015).

**Figure 3:**
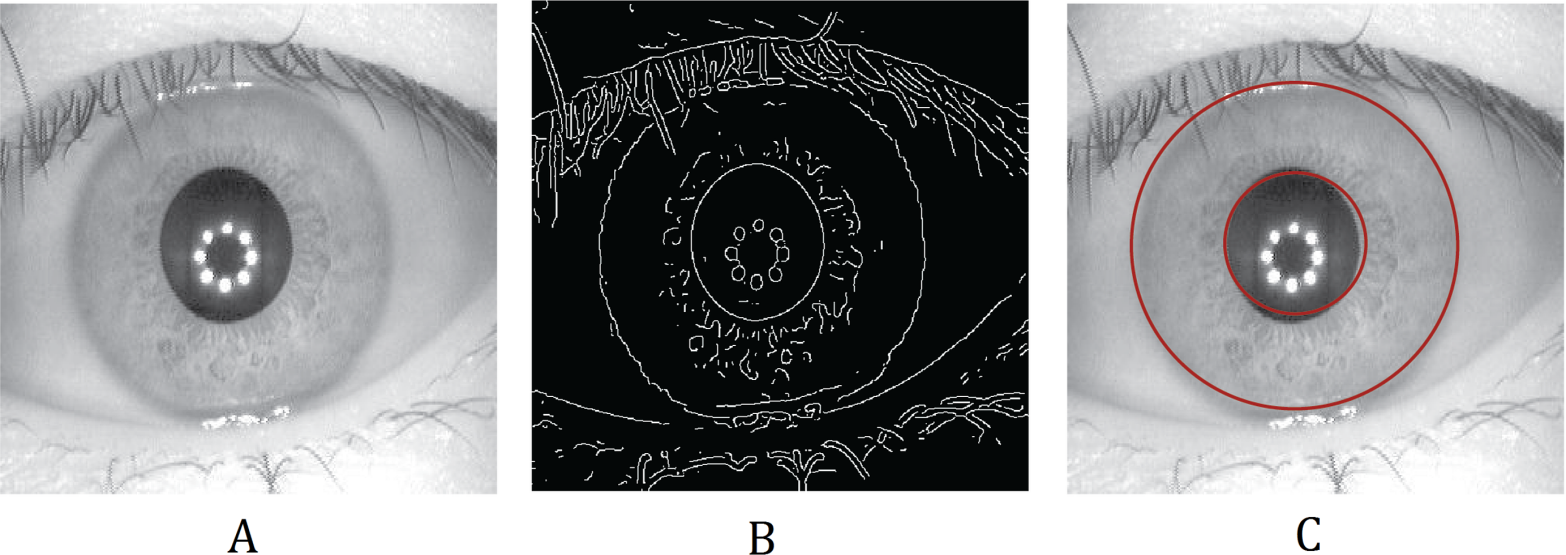
Iris segmentation using Hough transformation. (A) Original iris image; (B) edge mapped iris using first derivative or gradient; (C) edge detected iris image.

### Iris segmentation

Iris segmentation was used to isolate the actual iris region from a digital eye image. The iris region, (Fig. 1) can be bounded by two circles pertaining to the pupil (inner boundary) and sclera (outer boundary). Hough Transformation was employed to locate the circular iris region.

#### Hough transformation

The Hough transformation is a procedure generally used to compute the parameters of the geometric objects such as lines and circles in an image. For detecting the center coordinates and radius of the iris and pupil regions, the circular Hough transform can be used. This technique generally uses a voting procedure to find shapes of the objects within the classes available. The Hough segmentation algorithm firstly creates an edge map by computing the gradients or first derivatives of intensity values in an eye image as shown in Fig. 3. For each edge pixel in the edge map, the surrounding points on the circle at different radii are taken, and votes are cast for finding the maximum values that constitute the parameters of circles in the Hough space (Verma et al., 2012a). The center coordinates and the radius can be found using the following equation:

(1) \begin{eqnarray*}{x}_{c}^{2}+{y}_{c}^{2}-{r}^{2}=0\end{eqnarray*}

In the Hough space, the maximum point corresponds to the center coordinates (*x*_*c*_, *y*_*c*_) and the radius ‘r’ of the circle is given by the edge points.

When performing the edge detection, we have considered derivatives/gradients in the vertical direction to detect the iris-sclera boundary to decrease the effect of the eyelids which are horizontally aligned (Verma et al., 2012a). The vertical gradients are taken for locating the iris boundary and to reduce the influence of the eyelids. When performing circular Hough Transformation, not all of the edge pixels that define the circle are required for successful localization. Not only does this make circle localization more accurate, but it also makes it more efficient, since there are fewer edge points to cast votes in the Hough space (Verma et al., 2012a).

### Normalization

Once the circular iris region is successfully segmented from an eye image, normalization is applied on it to transform the segmented circular iris region into a fixed size rectangular shape. The normalization process produces an iris region that has fixed dimensions so that two photographs of the same iris taken under the different capturing environment will have the same characteristic features (Verma et al., 2012b). In this work, Daugman’s rubber-sheet model was used to normalize iris image.

#### Daugman’s rubber-sheet model

Daugman’s rubber-sheet model is the most widely used method for iris normalization (Daugman, 1993) which converts the circular iris region into a fixed sized rectangular block. Using (2), the model transforms every pixel in the circular iris into an equivalent position on the polar axes (*r*, *θ*) where r is the radial distance and *θ* is the rotated angle at the corresponding radius. The radial resolution describes the number of radial lines generated around the iris region while the angular resolution is the number of data points in the radial direction.

(2) \begin{eqnarray*}I[x(r,\theta ),y(r,\theta )]\rightarrow I(r,\theta )\end{eqnarray*}

**Figure 4:**
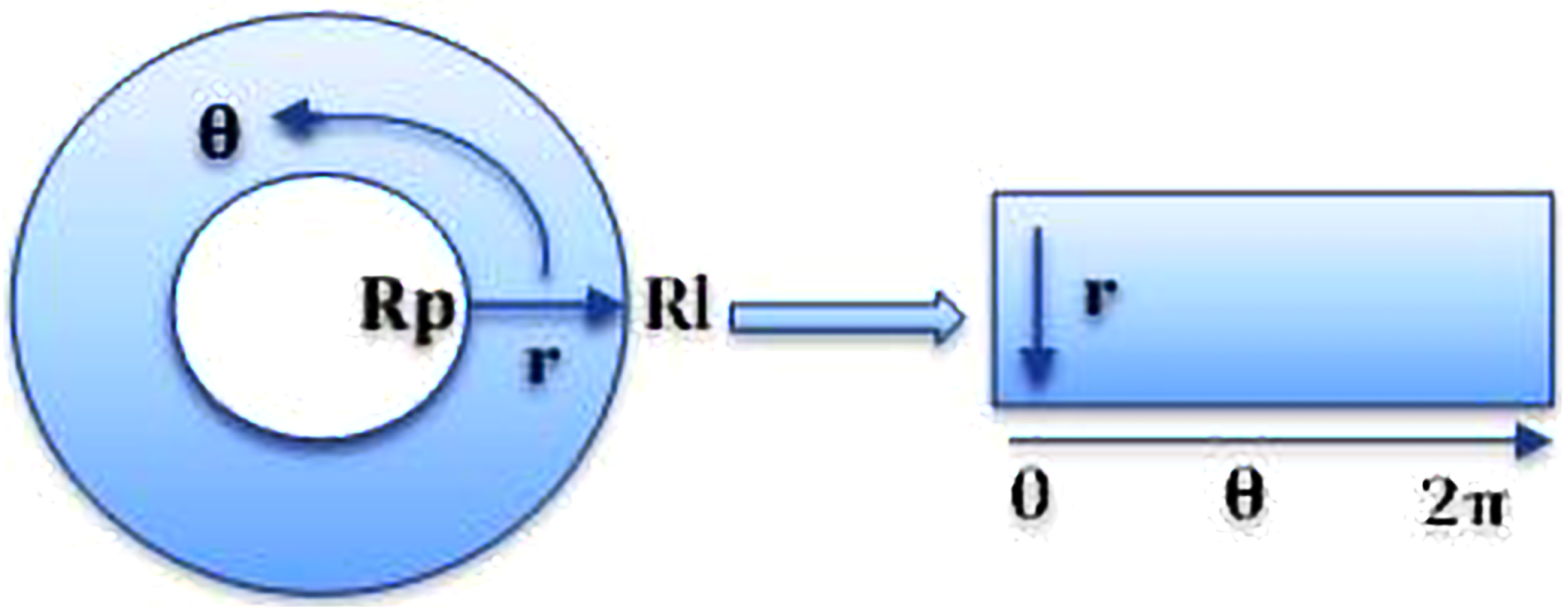
Daugman’s rubber-sheet model used for iris normalization. The circular shape represents segmented iris region and the rectangular shape represents normalized iris which is equivalent to the segmented iris region.

The iris region is converted to a two-dimensional array. Horizontal dimension represents the angular resolution, and the vertical dimension represents radial resolution, as shown in Fig. 4.

Where *I*(*x*, *y*) corresponds to the iris region, (*x*, *y*) and (*r*, *θ*) are the Cartesian and normalized polar coordinates, respectively. Ranges of *θ* from 0 to 2*π* and *r* from *Rp* to *Rl*.*x*(*r*, *θ*) and *y*(*r*, *θ*) are defined as linear combinations of pupil boundary points (Daugman, 1993).

### Feature extraction

Feature extraction is the most important and critical part of the iris recognition system. Feature extraction is a process of reducing the amount of data required to describe a large set of information present in an iris pattern. The successful recognition rate and reduction of classification time of two iris templates mostly depend on efficient feature extraction technique.

#### Proposed technique for feature extraction

**Figure 5:**
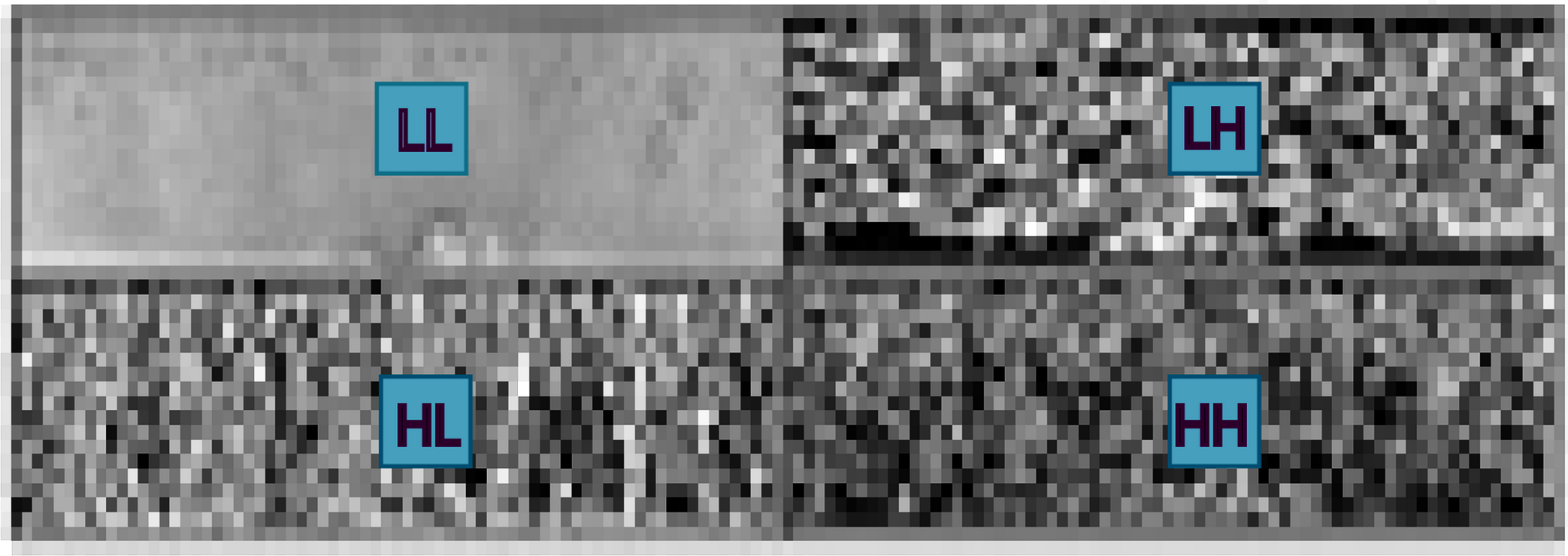
Iris template using one level DWT. DWT transforms normalized iris image into four-frequency sub-bands, namely LL, LH, HL and HH.

**Figure 6:**
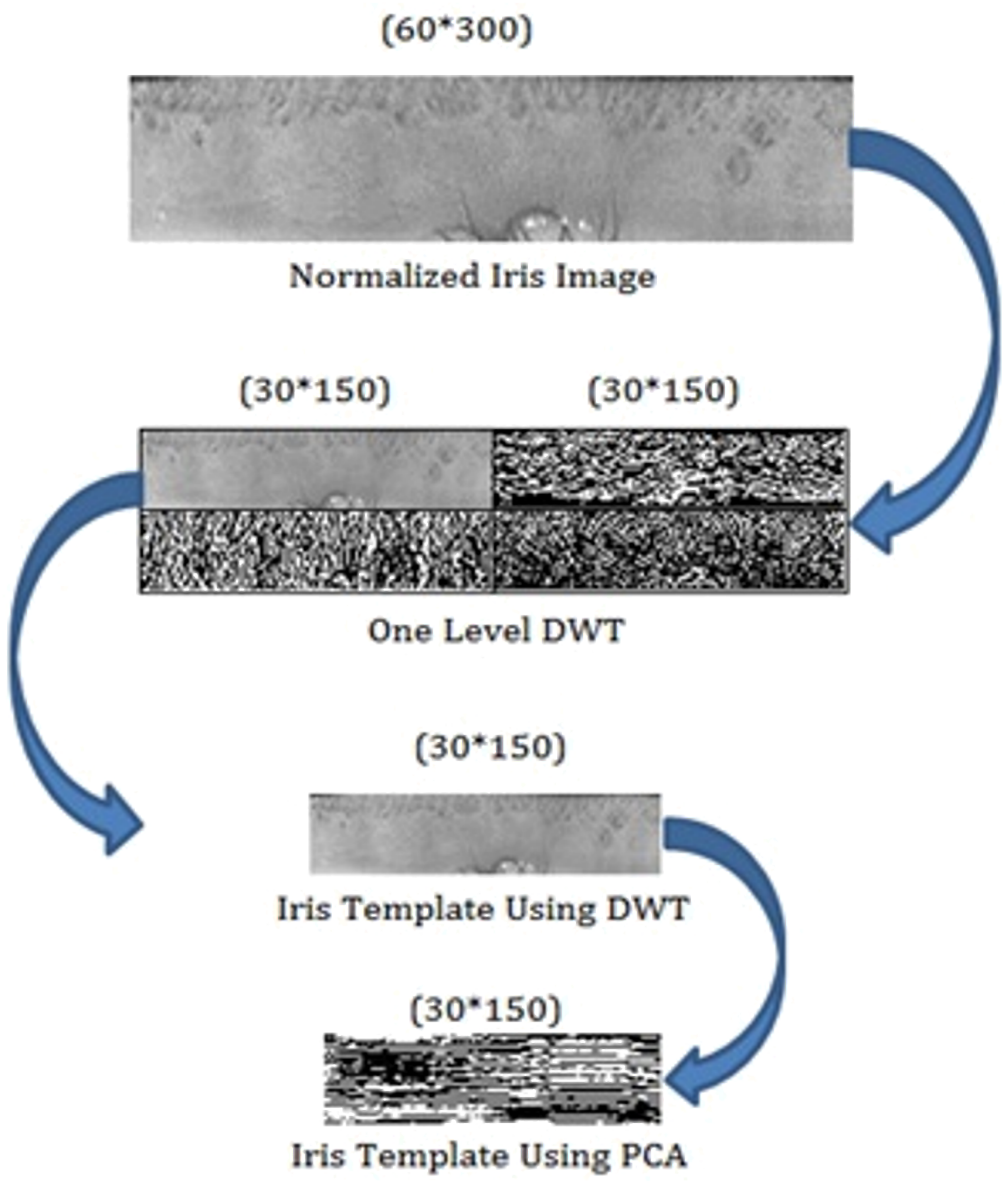
Iris template using PCA based on the DWT.

In this section, the proposed technique produces an iris template with reduced resolution and runtime for classifying the iris templates. To produce the template, firstly DWT has been applied to the normalized iris image. DWT transforms normalized iris image into four-frequency sub-bands, namely LL, LH, HL and HH, as shown in Fig. 5. The frequency range can be represented as LL < LH < HL < HH (Ma et al., 2004; Yu-Hsiang, 0000). The LL sub-band represents the feature or characteristics of the iris (Acharya et al., 2017; Moni & Ali, 2009), so that this sub-band can be considered for further processing (Acharya et al., 2017; Zhao et al., 2018). Figure 6 shows that the resolution of the original normalized iris image is (60 × 300). After applying DWT on a normalized iris image the resolution of LL sub-band is (30 × 150). LL sub-band represents the lower resolution approximation iris with required feature or characteristics, as this sub-band has been used instead of the original normalized iris data for further processing using PCA. As the resolution of the iris template has been reduced, so the runtime of the classification will be similarly reduced.

PCA has found the most discriminating information presented in LL sub-band to form feature matrix shown in Fig. 6, and the resultant feature matrix has been passed into the classifier for recognition.

The mathematical analysis of PCA includes:

1. The mean of each vector is given in the equation:
  (3) \begin{eqnarray*}{x}_{m}= \frac{1}{N} \sum _{k=1}^{N}xk\end{eqnarray*}
2. The mean is subtracted from all of the vectors to produce a set of zero mean vectors is given in the equation:
  (4) \begin{eqnarray*}{x}_{z}={x}_{i}-{x}_{m}\end{eqnarray*}
  where *x*_*z*_ is the zero mean vectors, *x*_*i*_ is each element of the column vector, *x*_*m*_ is the mean of each column vector.
3. The Covariance matrix is computed using the equation:
  (5) \begin{eqnarray*}c=[{x}_{z}T\ast {x}_{z}]\end{eqnarray*}
4. The Eigenvectors and Eigenvalues are computed using the equation:
  (6) \begin{eqnarray*}[c-\gamma i]e=0\end{eqnarray*}
  where *γ*’s are the Eigenvalue and e’s are the Eigenvectors.
5. Each of an Eigenvectors is multiplied with zero mean vectors *x*_*z*_ to form the feature vector. The feature vector is given by the equation:
  (7) \begin{eqnarray*}{f}_{i}=[{x}_{z}]e\end{eqnarray*}

### Classification

Classification is a process of measuring the similarity between two iris templates that have been generated in the feature extraction stage. This process gives a range of values during comparison of same iris templates and another range of values during the comparison of different iris templates generated from a different person’s eye (Rana, Azam & Akhtar, 2017). This training can ultimately enable us to determine whether the two iris templates belong to the same or different persons. In this work, Support Vector Machine was used to classify the iris images.

#### Support vector machine

**Figure 7:**
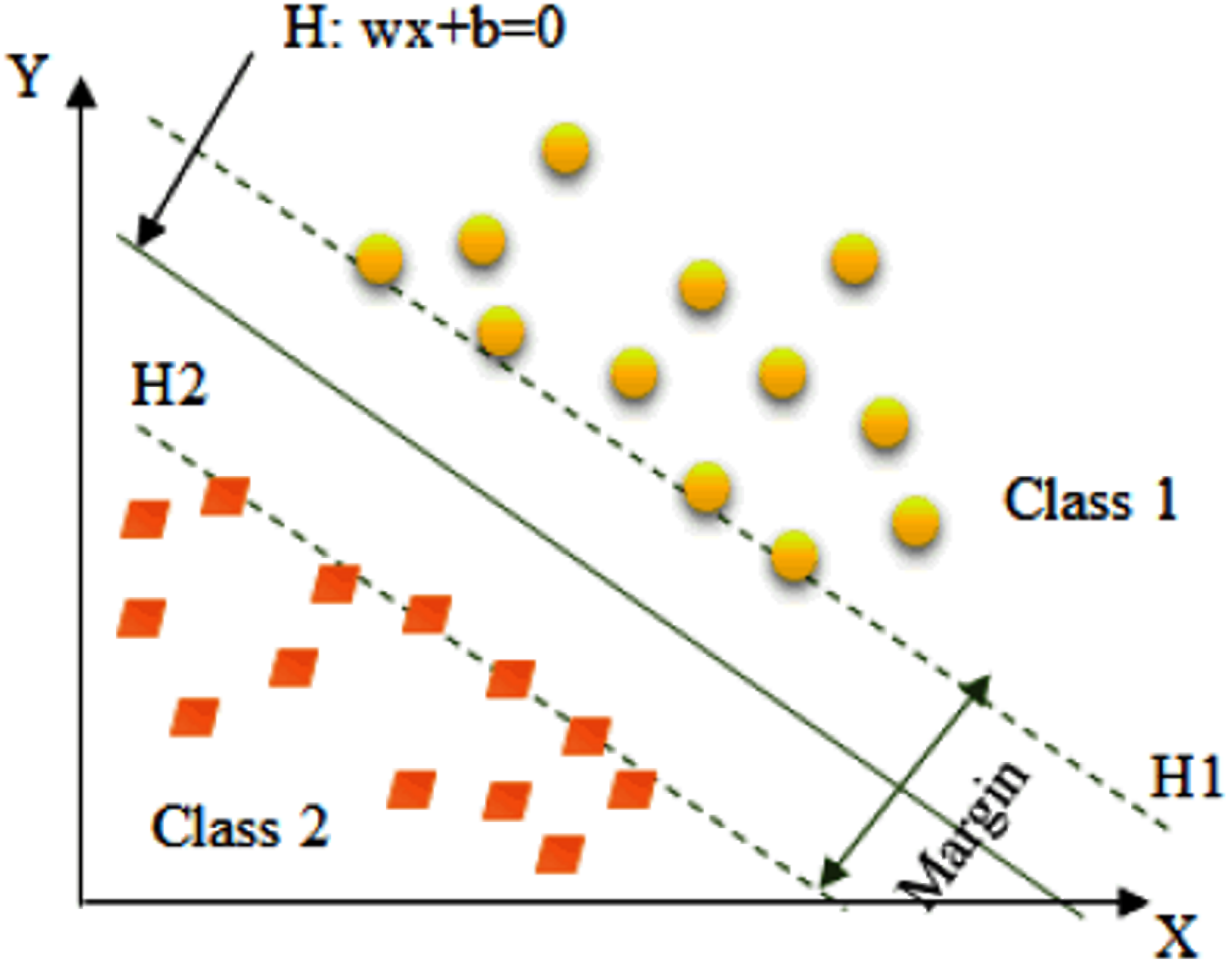
SVM with Linear separable data. The SVM finds the hyper-plane H (w.x+b=0) that differentiates the two classes of linear separable data.

Support vector machine (SVM) performs pattern recognition based on the principle of structural risk minimization. SVM is a binary classifier that optimally separates the two classes of data. There are two major aspects of developing SVM as a classifier. The first aspect is to determine the optimal hyperplane in between two separate classes of data and the another aspect is to transform the non-linearly separable classification problem into linearly separable problem (Czajka et al., 2017). Figure 7 shows an example of Linearly separable classification problem.

Let *x* is a set of input feature vector and *y* is the class label. The input feature vectors and the class label can be represented as {*x*_*i*_, *y*_*i*_}, where *i* = 1, 2, …, *N* and *y* =  ± 1. The separating hyperplane can be represented as follows,

(8) \begin{eqnarray*}w.x+b=0\end{eqnarray*}

which implies,

(9) \begin{eqnarray*}{y}_{i}(w.{x}_{i}+b)\geq 1;i=1,2...N\end{eqnarray*}

Basically, {*w*, *b*} can have numerous possible values which create separating hyperplane. It is believed that points often lie between two data classes in such a way that there is always some margin in between them. SVM maximizes this margin by considering it as a quadratic problem (Barpanda et al., 2018). The SVM can make two possible decisions during iris recognition; acceptance or rejection of a person.

## Algorithm

**Problem Definition:** The iris is a biometric characteristic that can be used to authenticate persons. This algorithm finds whether a person is authenticated or not using the iris.

**The objectives are:** This algorithm recognizes a person using iris segmentation, normalization, DWT, PCA and SVM classifier is given in Table 1.

**Table 1:** General algorithm of our proposed iris recognition system.

Input: Eye image
Output: Recognition of a person
(a) Read the eye image.
(b) Iris segmentation using Hough Transformation.
(c) Iris normalization using Daugman’s model.
(d) The DWT is applied, and the LL sub-band is considered.
(e) PCA is applied on LL sub-band to form a feature vector.
(f) Classification time measurement started by using a clock function.
(f) Match/Non-match decision is obtained using SVM classifier.
(g) Classification time measurement end.

## Experimental Results and Discussions

In this section, the proposed technique has been evaluated in the CASIA iris database, and the results have been reported. 100 iris images of 100 individuals have been enrolled in our system database for training and other 100 iris images for assessing the performance. Intel Core-i5 3.30GH processor, 4GB RAM, Windows-7 operating system and MATLAB2017a tools have been used as an experimental environment. In this study, we have developed an approach to reduce the runtime by keeping the accuracy as high as possible. The accuracy of our proposed technique is 95.4% with FAR 4% and FRR 5%, is shown in Table 2, which is better than the other methods that are used for runtime comparison.

**Table 2:** Accuracy comparison of our proposed technique with others.

Sl.	Author	Method	Accuracy
1.	MH Hamd et al.	PCA	94%
2.	SG Firake et al.	Gabor filter	92.85%
3.	J poonia et al.	Gabor + PCA	82.5%
4.	HK Rana et al.	Our proposed	95.4%

**Table 3:** Comparison of classification-time of our proposed technique with others. Runtime for each feature extraction technique has been reported by employing 100 iris templates. The mean and median of each feature extraction technique have been calculated by considering the runtime of eight attempts.

Sl.	Methods	Runtime of 100 iris templates in second		
		Time per attempt	Mean	Median
1.	PCA based feature extraction	11.0352	11.0354	11.0345
		11.0338		
		11.0573		
		11.0192		
		11.0345		
		11.0216		
		11.0466		
		11.0573		
2.	Gabor filter based feature extraction	15.3509	15.3100	15.3112
		15.2496		
		15.2808		
		15.2908		
		15.2485		
		15.3780		
		15.3501		
		15.3315		
3.	Gabor filter + PCA based feature extraction	13.0499	12.9525	12.9363
		12.9177		
		12.9376		
		12.9349		
		12.9676		
		12.9880		
		12.9019		
		12.9225		
4.	Proposed feature extraction technique	9.1325	9.1598	9.1471
		9.1135		
		9.1605		
		9.2205		
		9.1969		
		9.1229		
		9.1337		
		9.1982		

Hamd & Ahmed (2018) proposed a technique to compare two feature extraction methods, PCA and Fourier descriptors (FD), in which circular Hough transform, Daugman’s rubber sheet model and the Manhattan classifier were used for segmentation, normalization, and classification respectively. Their average accuracy for PCA was 94%. Firake & Mahajan (2016) proposed a technique to compare three feature extraction methods, Gabor filter, PCA and independent component analysis (ICA), in which Hough transform, Daugman’s rubber sheet model and Hamming distance were used for segmentation, normalization, and classification respectively. Their average accuracy was 92.85% for Gabor filter. Jyoti poonia (2016) observed the average accuracy 82.5% for Gabor filter + PCA based feature extraction technique.

**Figure 8:**
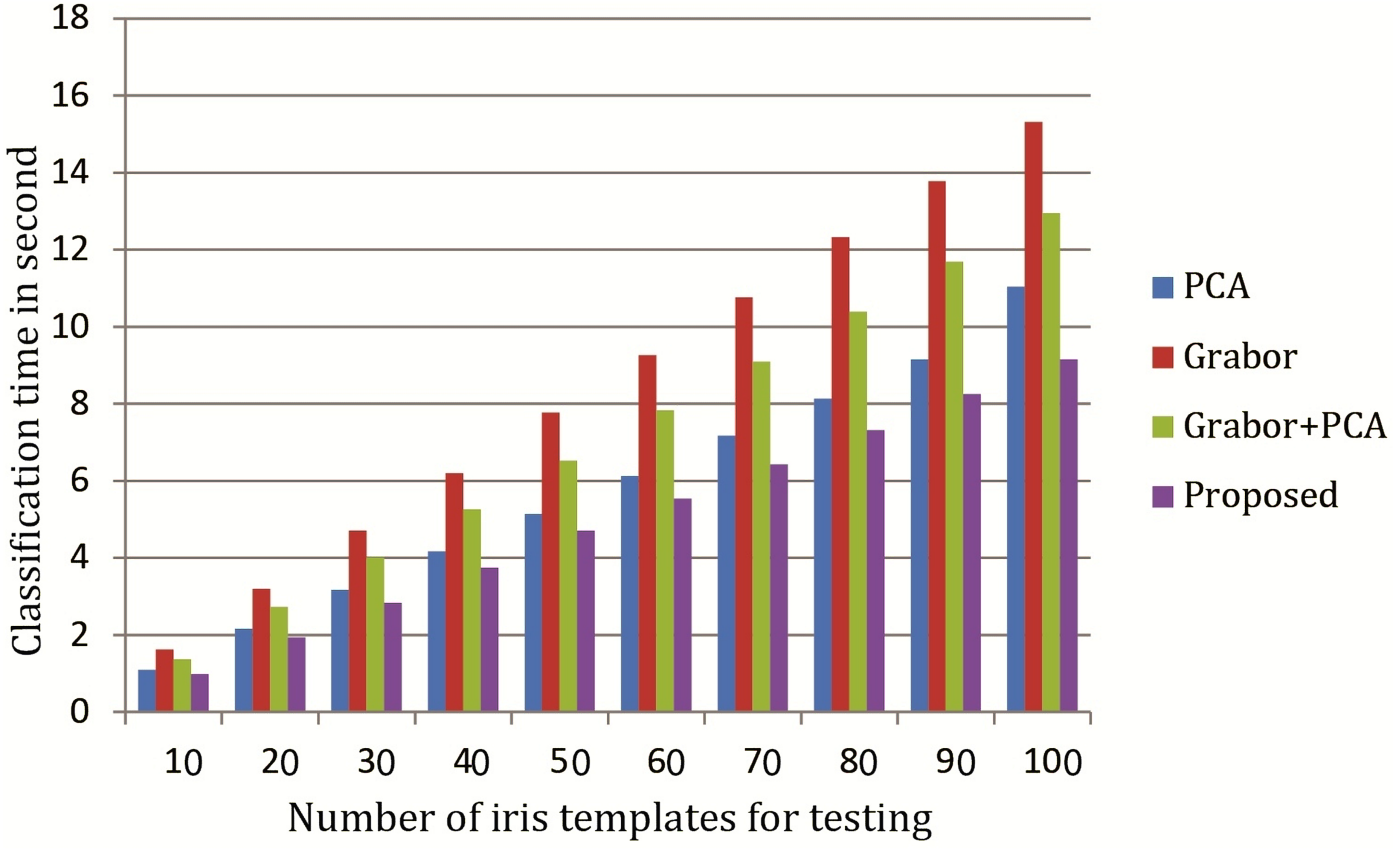
Comparison of classification-time of our proposed technique with varying testing samples.

We have mainly evaluated the time required for classification of our proposed feature extraction techniques, and found classification-time of our proposed technique is significantly better than others. The experimental results are shown in Table 3 and Fig. 8.

## Conclusions

In this paper, we proposed a technique that used Principal Component Analysis (PCA) based on Discrete Wavelet Transformation (DWT) for extracting the optimum features of iris images and reducing the runtime of classification of these iris templates. The point of using DWT behind PCA is to reduce the iris template resolution. DWT converts the iris image into four frequency bands, and one frequency band instead of four has been used for further feature extraction by using PCA. As the resolution of iris template has been reduced by DWT, so the runtime of classification will be reduced. Experimental evaluation using the CASIA iris image database (ver. 4) clearly demonstrated the proposed technique performs in a very efficient manner.

##  Supplemental Information

10.7717/peerj-cs.184/supp-1 Supplemental Information 1 Data and code

## Additional Information and Declarations

### Data Availability

The following information was supplied regarding data availability: The codes and raw data are available in the Supplemental Files.
