# Supplementary figures and images for: A fast iris recognition system through optimum feature extraction (part 2 of 2)
Source: PeerJ Comput Sci. 2019 Apr 8;5:e184. doi: 10.7717/peerj-cs.184 (PMC7924705; doi:10.7717/peerj-cs.184)

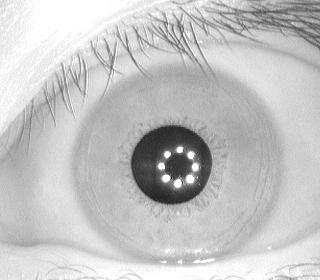

Supplement: Supplemental Information 1 [file peerj-cs-05-184-s001.zip › code9 PeerJ/Part1/gallery/g95.jpg]

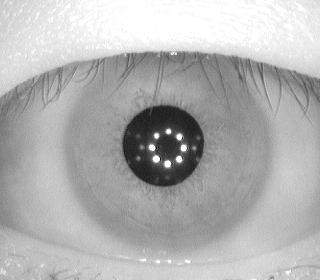

Supplement: Supplemental Information 1 [file peerj-cs-05-184-s001.zip › code9 PeerJ/Part1/gallery/g96.jpg]

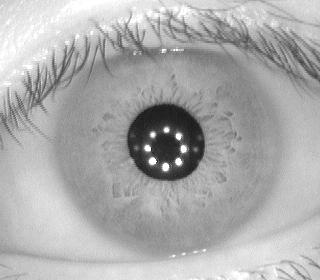

Supplement: Supplemental Information 1 [file peerj-cs-05-184-s001.zip › code9 PeerJ/Part1/gallery/g97.jpg]

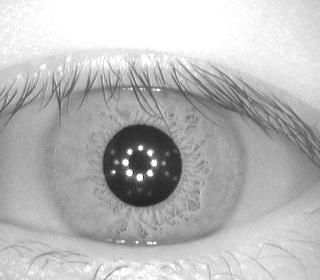

Supplement: Supplemental Information 1 [file peerj-cs-05-184-s001.zip › code9 PeerJ/Part1/gallery/g98.jpg]

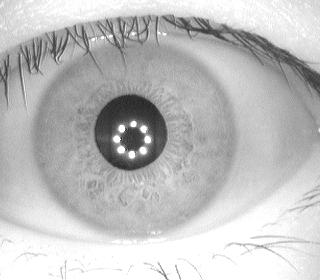

Supplement: Supplemental Information 1 [file peerj-cs-05-184-s001.zip › code9 PeerJ/Part1/gallery2/g1/g110.jpg]

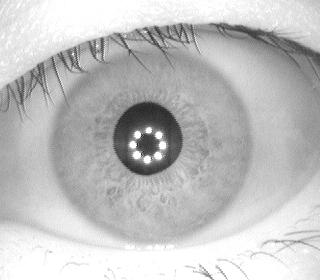

Supplement: Supplemental Information 1 [file peerj-cs-05-184-s001.zip › code9 PeerJ/Part1/gallery2/g1/g12.jpg]

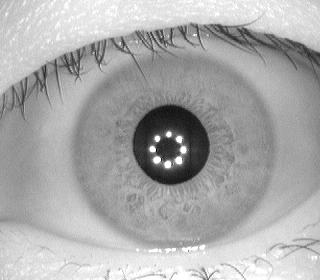

Supplement: Supplemental Information 1 [file peerj-cs-05-184-s001.zip › code9 PeerJ/Part1/gallery2/g1/g13.jpg]

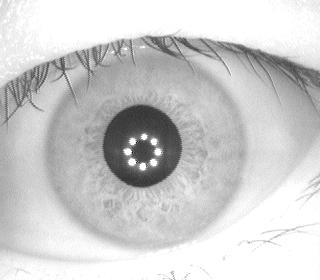

Supplement: Supplemental Information 1 [file peerj-cs-05-184-s001.zip › code9 PeerJ/Part1/gallery2/g1/g14.jpg]

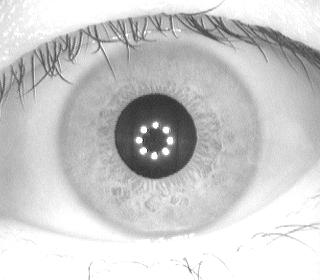

Supplement: Supplemental Information 1 [file peerj-cs-05-184-s001.zip › code9 PeerJ/Part1/gallery2/g1/g15.jpg]

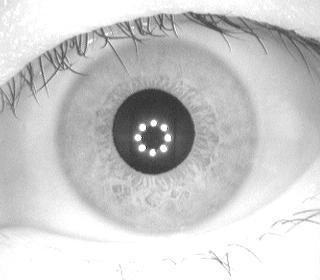

Supplement: Supplemental Information 1 [file peerj-cs-05-184-s001.zip › code9 PeerJ/Part1/gallery2/g1/g16.jpg]

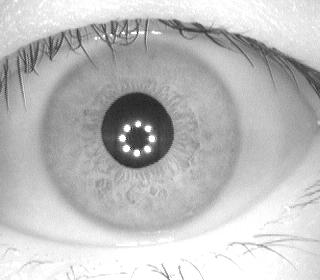

Supplement: Supplemental Information 1 [file peerj-cs-05-184-s001.zip › code9 PeerJ/Part1/gallery2/g1/g17.jpg]

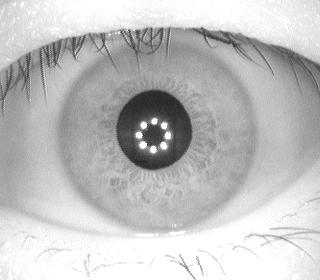

Supplement: Supplemental Information 1 [file peerj-cs-05-184-s001.zip › code9 PeerJ/Part1/gallery2/g1/g18.jpg]

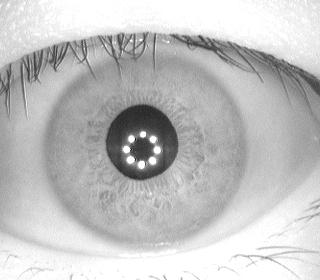

Supplement: Supplemental Information 1 [file peerj-cs-05-184-s001.zip › code9 PeerJ/Part1/gallery2/g1/g19.jpg]

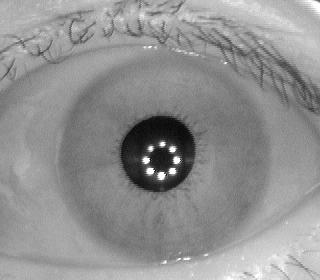

Supplement: Supplemental Information 1 [file peerj-cs-05-184-s001.zip › code9 PeerJ/Part1/gallery2/g2/g21.jpg]

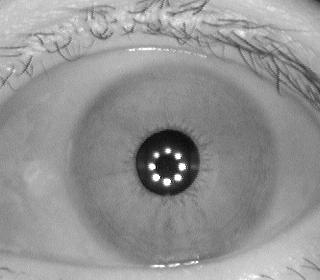

Supplement: Supplemental Information 1 [file peerj-cs-05-184-s001.zip › code9 PeerJ/Part1/gallery2/g2/g22.jpg]

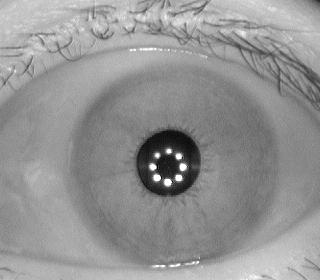

Supplement: Supplemental Information 1 [file peerj-cs-05-184-s001.zip › code9 PeerJ/Part1/gallery2/g2/g24.jpg]

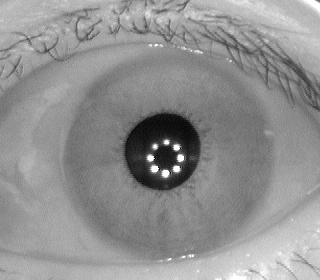

Supplement: Supplemental Information 1 [file peerj-cs-05-184-s001.zip › code9 PeerJ/Part1/gallery2/g2/g25.jpg]

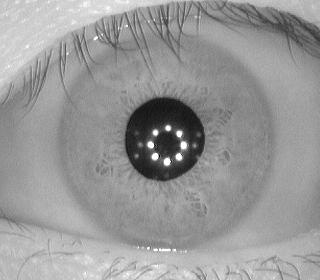

Supplement: Supplemental Information 1 [file peerj-cs-05-184-s001.zip › code9 PeerJ/Part1/gallery2/g6/g62.jpg]

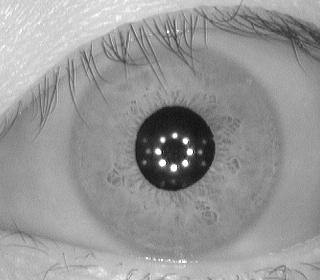

Supplement: Supplemental Information 1 [file peerj-cs-05-184-s001.zip › code9 PeerJ/Part1/gallery2/g6/g63.jpg]

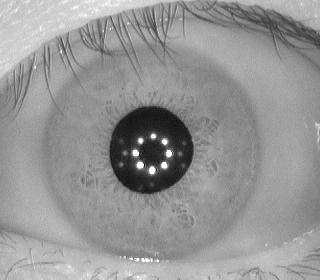

Supplement: Supplemental Information 1 [file peerj-cs-05-184-s001.zip › code9 PeerJ/Part1/gallery2/g6/g64.jpg]

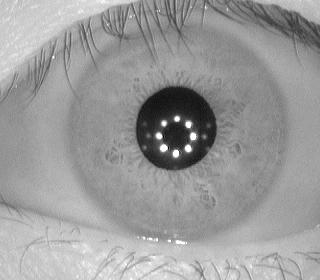

Supplement: Supplemental Information 1 [file peerj-cs-05-184-s001.zip › code9 PeerJ/Part1/gallery2/g6/g65.jpg]

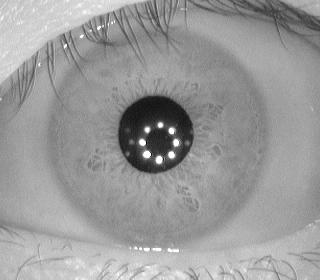

Supplement: Supplemental Information 1 [file peerj-cs-05-184-s001.zip › code9 PeerJ/Part1/gallery2/g6/g66.jpg]

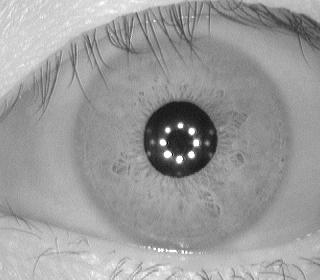

Supplement: Supplemental Information 1 [file peerj-cs-05-184-s001.zip › code9 PeerJ/Part1/gallery2/g6/g67.jpg]

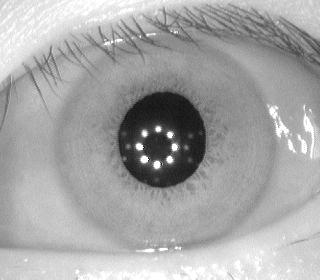

Supplement: Supplemental Information 1 [file peerj-cs-05-184-s001.zip › code9 PeerJ/Part1/gallery2/g8/g81.jpg]

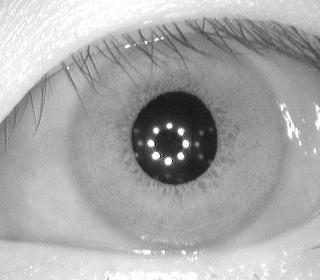

Supplement: Supplemental Information 1 [file peerj-cs-05-184-s001.zip › code9 PeerJ/Part1/gallery2/g8/g82.jpg]

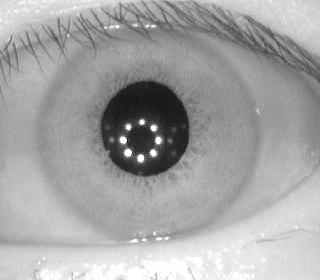

Supplement: Supplemental Information 1 [file peerj-cs-05-184-s001.zip › code9 PeerJ/Part1/gallery2/g8/g83.jpg]

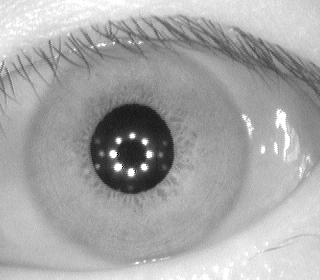

Supplement: Supplemental Information 1 [file peerj-cs-05-184-s001.zip › code9 PeerJ/Part1/gallery2/g8/g85.jpg]

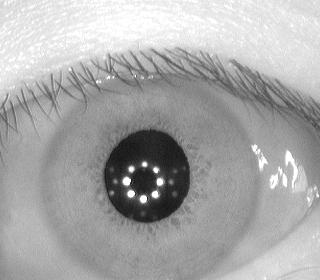

Supplement: Supplemental Information 1 [file peerj-cs-05-184-s001.zip › code9 PeerJ/Part1/gallery2/g8/g86.jpg]

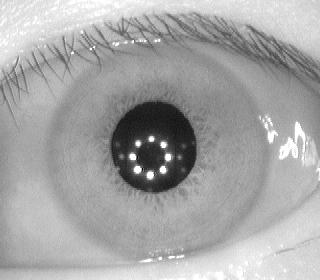

Supplement: Supplemental Information 1 [file peerj-cs-05-184-s001.zip › code9 PeerJ/Part1/gallery2/g8/g87.jpg]

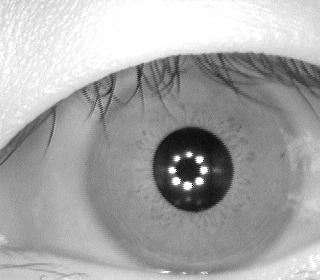

Supplement: Supplemental Information 1 [file peerj-cs-05-184-s001.zip › code9 PeerJ/Part1/sample/s2.jpg]

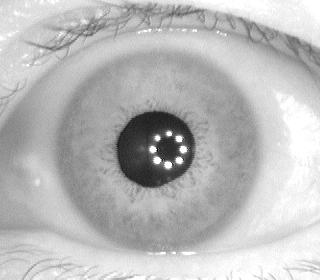

Supplement: Supplemental Information 1 [file peerj-cs-05-184-s001.zip › code9 PeerJ/Part1/sample/s3.jpg]

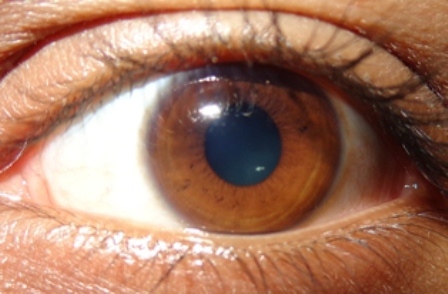

Supplement: Supplemental Information 1 [file peerj-cs-05-184-s001.zip › code9 PeerJ/Part1/sample/s4.jpg]

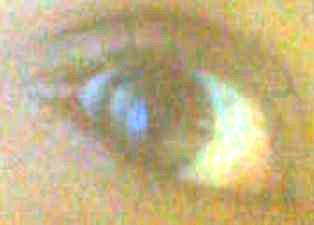

Supplement: Supplemental Information 1 [file peerj-cs-05-184-s001.zip › code9 PeerJ/Part1/sample/s6.jpg]

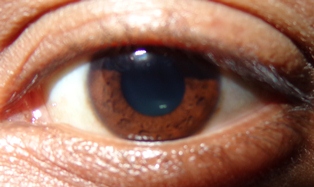

Supplement: Supplemental Information 1 [file peerj-cs-05-184-s001.zip › code9 PeerJ/Part1/sample/s7.jpg]

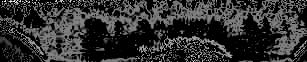

Supplement: Supplemental Information 1 [file peerj-cs-05-184-s001.zip › code9 PeerJ/Part2 (measuring runtime)/gabor/database/d1.jpg]

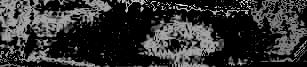

Supplement: Supplemental Information 1 [file peerj-cs-05-184-s001.zip › code9 PeerJ/Part2 (measuring runtime)/gabor/database/d10.jpg]

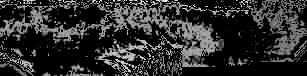

Supplement: Supplemental Information 1 [file peerj-cs-05-184-s001.zip › code9 PeerJ/Part2 (measuring runtime)/gabor/database/d11.jpg]

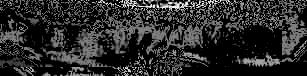

Supplement: Supplemental Information 1 [file peerj-cs-05-184-s001.zip › code9 PeerJ/Part2 (measuring runtime)/gabor/database/d12.jpg]

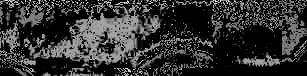

Supplement: Supplemental Information 1 [file peerj-cs-05-184-s001.zip › code9 PeerJ/Part2 (measuring runtime)/gabor/database/d13.jpg]

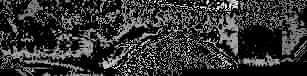

Supplement: Supplemental Information 1 [file peerj-cs-05-184-s001.zip › code9 PeerJ/Part2 (measuring runtime)/gabor/database/d14.jpg]

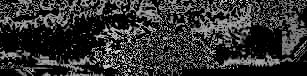

Supplement: Supplemental Information 1 [file peerj-cs-05-184-s001.zip › code9 PeerJ/Part2 (measuring runtime)/gabor/database/d15.jpg]

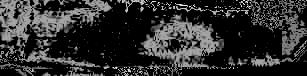

Supplement: Supplemental Information 1 [file peerj-cs-05-184-s001.zip › code9 PeerJ/Part2 (measuring runtime)/gabor/database/d16.jpg]

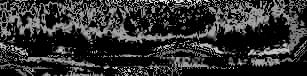

Supplement: Supplemental Information 1 [file peerj-cs-05-184-s001.zip › code9 PeerJ/Part2 (measuring runtime)/gabor/database/d17.jpg]

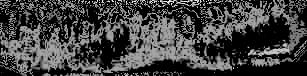

Supplement: Supplemental Information 1 [file peerj-cs-05-184-s001.zip › code9 PeerJ/Part2 (measuring runtime)/gabor/database/d18.jpg]

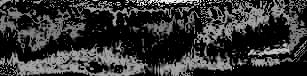

Supplement: Supplemental Information 1 [file peerj-cs-05-184-s001.zip › code9 PeerJ/Part2 (measuring runtime)/gabor/database/d19.jpg]

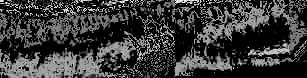

Supplement: Supplemental Information 1 [file peerj-cs-05-184-s001.zip › code9 PeerJ/Part2 (measuring runtime)/gabor/database/d2.jpg]

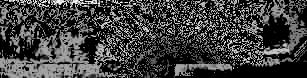

Supplement: Supplemental Information 1 [file peerj-cs-05-184-s001.zip › code9 PeerJ/Part2 (measuring runtime)/gabor/database/d20.jpg]

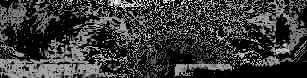

Supplement: Supplemental Information 1 [file peerj-cs-05-184-s001.zip › code9 PeerJ/Part2 (measuring runtime)/gabor/database/d21.jpg]

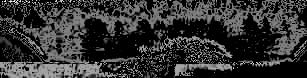

Supplement: Supplemental Information 1 [file peerj-cs-05-184-s001.zip › code9 PeerJ/Part2 (measuring runtime)/gabor/database/d22.jpg]

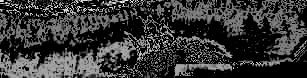

Supplement: Supplemental Information 1 [file peerj-cs-05-184-s001.zip › code9 PeerJ/Part2 (measuring runtime)/gabor/database/d23.jpg]

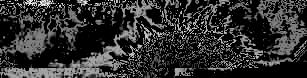

Supplement: Supplemental Information 1 [file peerj-cs-05-184-s001.zip › code9 PeerJ/Part2 (measuring runtime)/gabor/database/d24.jpg]

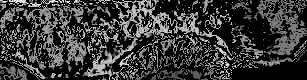

Supplement: Supplemental Information 1 [file peerj-cs-05-184-s001.zip › code9 PeerJ/Part2 (measuring runtime)/gabor/database/d25.jpg]

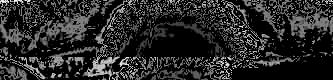

Supplement: Supplemental Information 1 [file peerj-cs-05-184-s001.zip › code9 PeerJ/Part2 (measuring runtime)/gabor/database/d26.jpg]

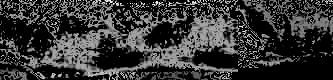

Supplement: Supplemental Information 1 [file peerj-cs-05-184-s001.zip › code9 PeerJ/Part2 (measuring runtime)/gabor/database/d27.jpg]

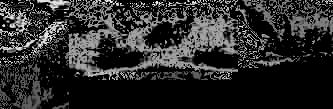

Supplement: Supplemental Information 1 [file peerj-cs-05-184-s001.zip › code9 PeerJ/Part2 (measuring runtime)/gabor/database/d28.jpg]

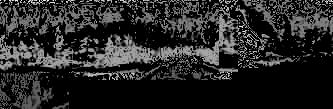

Supplement: Supplemental Information 1 [file peerj-cs-05-184-s001.zip › code9 PeerJ/Part2 (measuring runtime)/gabor/database/d29.jpg]

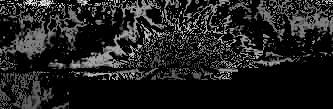

Supplement: Supplemental Information 1 [file peerj-cs-05-184-s001.zip › code9 PeerJ/Part2 (measuring runtime)/gabor/database/d3.jpg]

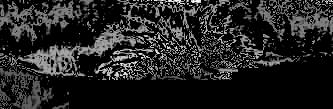

Supplement: Supplemental Information 1 [file peerj-cs-05-184-s001.zip › code9 PeerJ/Part2 (measuring runtime)/gabor/database/d30.jpg]

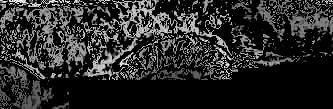

Supplement: Supplemental Information 1 [file peerj-cs-05-184-s001.zip › code9 PeerJ/Part2 (measuring runtime)/gabor/database/d4.jpg]

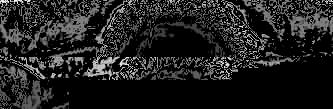

Supplement: Supplemental Information 1 [file peerj-cs-05-184-s001.zip › code9 PeerJ/Part2 (measuring runtime)/gabor/database/d5.jpg]

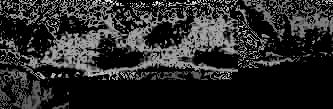

Supplement: Supplemental Information 1 [file peerj-cs-05-184-s001.zip › code9 PeerJ/Part2 (measuring runtime)/gabor/database/d6.jpg]

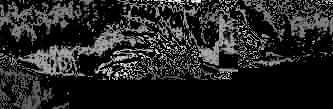

Supplement: Supplemental Information 1 [file peerj-cs-05-184-s001.zip › code9 PeerJ/Part2 (measuring runtime)/gabor/database/d9.jpg]
