# Supplementary figures and images for: A fast iris recognition system through optimum feature extraction (part 1 of 2)
Source: PeerJ Comput Sci. 2019 Apr 8;5:e184. doi: 10.7717/peerj-cs.184 (PMC7924705; doi:10.7717/peerj-cs.184)

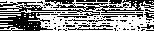

Supplement: Supplemental Information 1 [file peerj-cs-05-184-s001.zip › code9 PeerJ/Part1/database/d1.jpg]

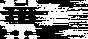

Supplement: Supplemental Information 1 [file peerj-cs-05-184-s001.zip › code9 PeerJ/Part1/database/d2.jpg]

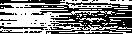

Supplement: Supplemental Information 1 [file peerj-cs-05-184-s001.zip › code9 PeerJ/Part1/database/d3.jpg]

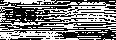

Supplement: Supplemental Information 1 [file peerj-cs-05-184-s001.zip › code9 PeerJ/Part1/database/d4.jpg]

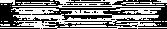

Supplement: Supplemental Information 1 [file peerj-cs-05-184-s001.zip › code9 PeerJ/Part1/database/d5.jpg]

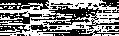

Supplement: Supplemental Information 1 [file peerj-cs-05-184-s001.zip › code9 PeerJ/Part1/database/d6.jpg]

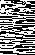

Supplement: Supplemental Information 1 [file peerj-cs-05-184-s001.zip › code9 PeerJ/Part1/database/d7.jpg]

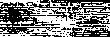

Supplement: Supplemental Information 1 [file peerj-cs-05-184-s001.zip › code9 PeerJ/Part1/database/d8.jpg]

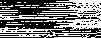

Supplement: Supplemental Information 1 [file peerj-cs-05-184-s001.zip › code9 PeerJ/Part1/database/d9.jpg]

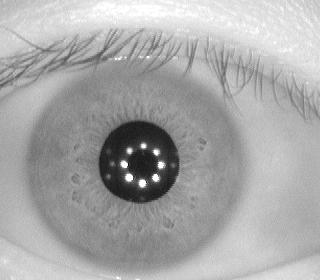

Supplement: Supplemental Information 1 [file peerj-cs-05-184-s001.zip › code9 PeerJ/Part1/g3.jpg]

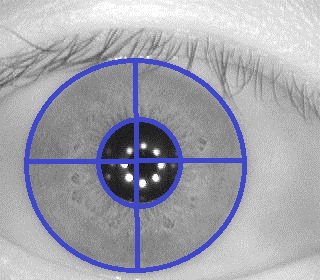

Supplement: Supplemental Information 1 [file peerj-cs-05-184-s001.zip › code9 PeerJ/Part1/g4.jpg]

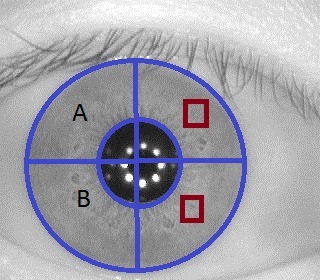

Supplement: Supplemental Information 1 [file peerj-cs-05-184-s001.zip › code9 PeerJ/Part1/g5.jpg]

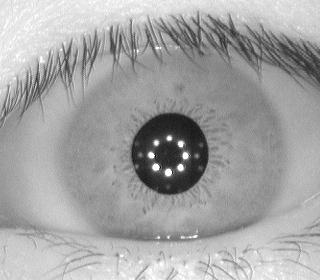

Supplement: Supplemental Information 1 [file peerj-cs-05-184-s001.zip › code9 PeerJ/Part1/gallery/g1.jpg]

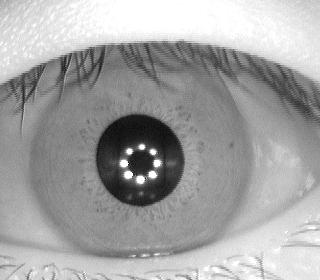

Supplement: Supplemental Information 1 [file peerj-cs-05-184-s001.zip › code9 PeerJ/Part1/gallery/g10.jpg]

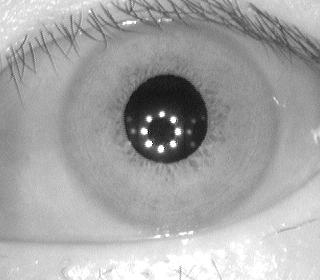

Supplement: Supplemental Information 1 [file peerj-cs-05-184-s001.zip › code9 PeerJ/Part1/gallery/g100.jpg]

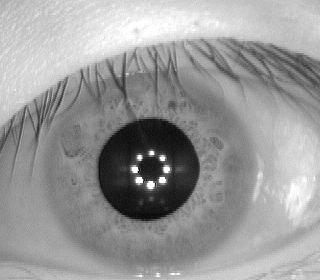

Supplement: Supplemental Information 1 [file peerj-cs-05-184-s001.zip › code9 PeerJ/Part1/gallery/g11.jpg]

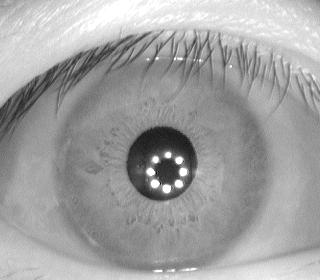

Supplement: Supplemental Information 1 [file peerj-cs-05-184-s001.zip › code9 PeerJ/Part1/gallery/g14.jpg]

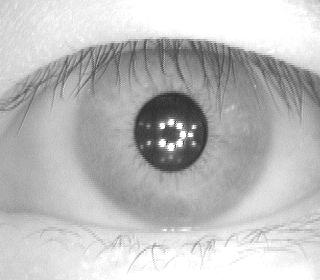

Supplement: Supplemental Information 1 [file peerj-cs-05-184-s001.zip › code9 PeerJ/Part1/gallery/g15.jpg]

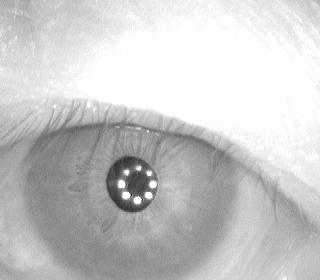

Supplement: Supplemental Information 1 [file peerj-cs-05-184-s001.zip › code9 PeerJ/Part1/gallery/g16.jpg]

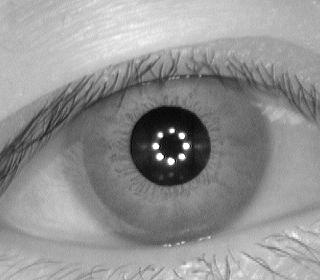

Supplement: Supplemental Information 1 [file peerj-cs-05-184-s001.zip › code9 PeerJ/Part1/gallery/g17.jpg]

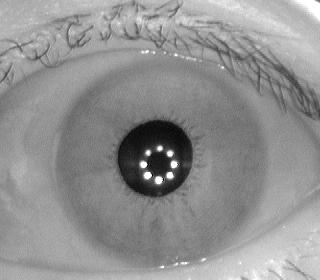

Supplement: Supplemental Information 1 [file peerj-cs-05-184-s001.zip › code9 PeerJ/Part1/gallery/g18.jpg]

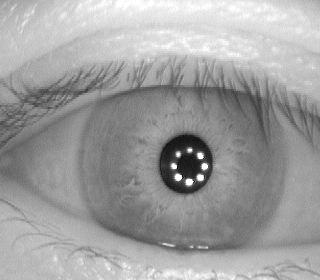

Supplement: Supplemental Information 1 [file peerj-cs-05-184-s001.zip › code9 PeerJ/Part1/gallery/g19.jpg]

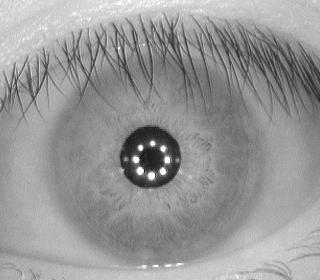

Supplement: Supplemental Information 1 [file peerj-cs-05-184-s001.zip › code9 PeerJ/Part1/gallery/g2.jpg]

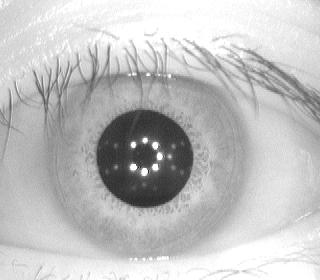

Supplement: Supplemental Information 1 [file peerj-cs-05-184-s001.zip › code9 PeerJ/Part1/gallery/g20.jpg]

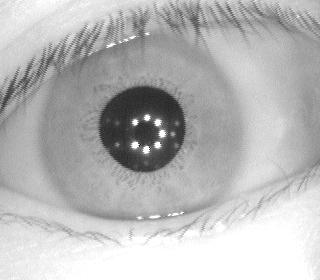

Supplement: Supplemental Information 1 [file peerj-cs-05-184-s001.zip › code9 PeerJ/Part1/gallery/g21.jpg]

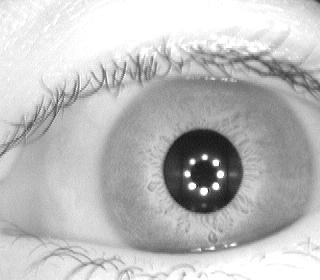

Supplement: Supplemental Information 1 [file peerj-cs-05-184-s001.zip › code9 PeerJ/Part1/gallery/g22.jpg]

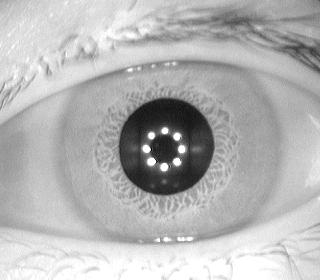

Supplement: Supplemental Information 1 [file peerj-cs-05-184-s001.zip › code9 PeerJ/Part1/gallery/g23.jpg]

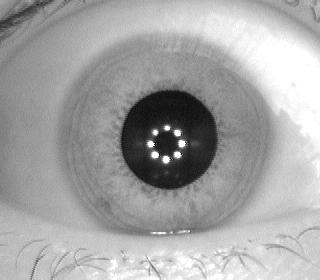

Supplement: Supplemental Information 1 [file peerj-cs-05-184-s001.zip › code9 PeerJ/Part1/gallery/g24.jpg]

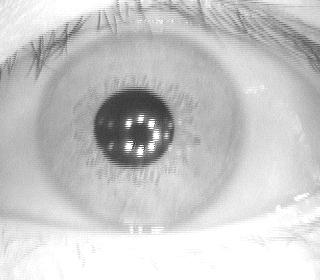

Supplement: Supplemental Information 1 [file peerj-cs-05-184-s001.zip › code9 PeerJ/Part1/gallery/g25.jpg]

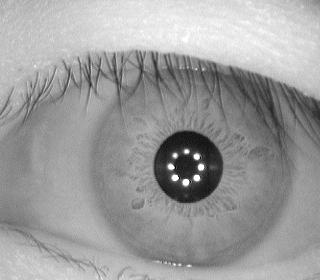

Supplement: Supplemental Information 1 [file peerj-cs-05-184-s001.zip › code9 PeerJ/Part1/gallery/g28.jpg]

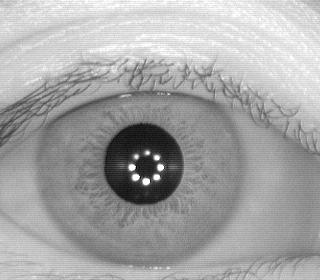

Supplement: Supplemental Information 1 [file peerj-cs-05-184-s001.zip › code9 PeerJ/Part1/gallery/g29.jpg]

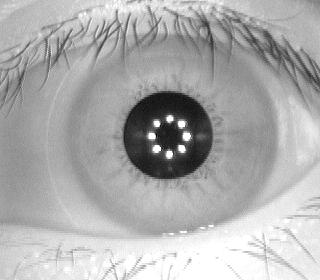

Supplement: Supplemental Information 1 [file peerj-cs-05-184-s001.zip › code9 PeerJ/Part1/gallery/g3.jpg]

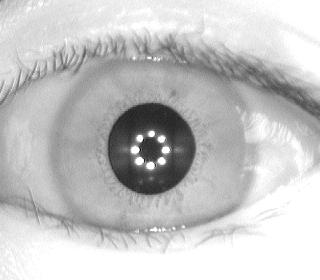

Supplement: Supplemental Information 1 [file peerj-cs-05-184-s001.zip › code9 PeerJ/Part1/gallery/g30.jpg]

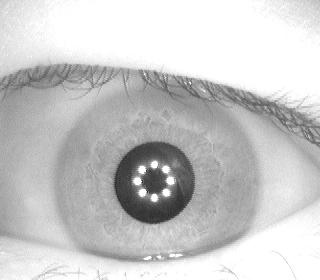

Supplement: Supplemental Information 1 [file peerj-cs-05-184-s001.zip › code9 PeerJ/Part1/gallery/g32.jpg]

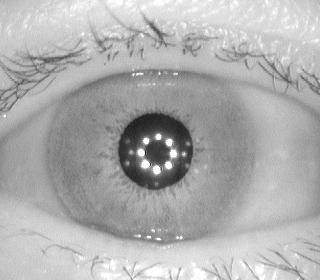

Supplement: Supplemental Information 1 [file peerj-cs-05-184-s001.zip › code9 PeerJ/Part1/gallery/g33.jpg]

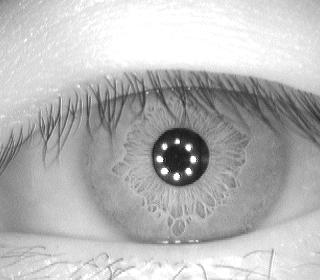

Supplement: Supplemental Information 1 [file peerj-cs-05-184-s001.zip › code9 PeerJ/Part1/gallery/g34.jpg]

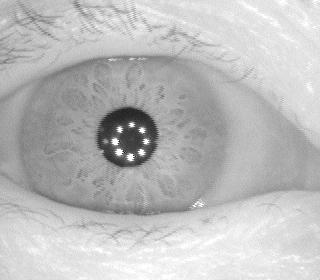

Supplement: Supplemental Information 1 [file peerj-cs-05-184-s001.zip › code9 PeerJ/Part1/gallery/g35.jpg]

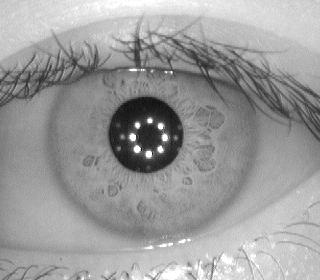

Supplement: Supplemental Information 1 [file peerj-cs-05-184-s001.zip › code9 PeerJ/Part1/gallery/g36.jpg]

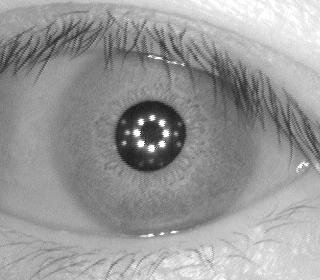

Supplement: Supplemental Information 1 [file peerj-cs-05-184-s001.zip › code9 PeerJ/Part1/gallery/g37.jpg]

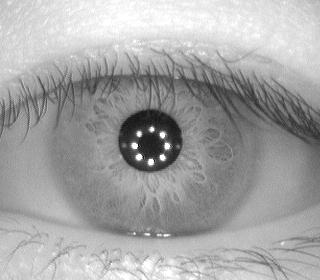

Supplement: Supplemental Information 1 [file peerj-cs-05-184-s001.zip › code9 PeerJ/Part1/gallery/g38.jpg]

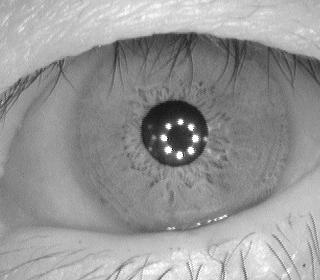

Supplement: Supplemental Information 1 [file peerj-cs-05-184-s001.zip › code9 PeerJ/Part1/gallery/g39.jpg]

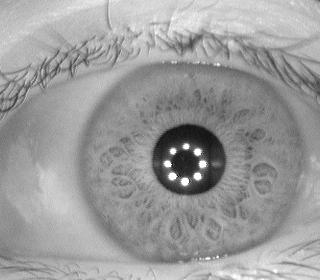

Supplement: Supplemental Information 1 [file peerj-cs-05-184-s001.zip › code9 PeerJ/Part1/gallery/g4.jpg]

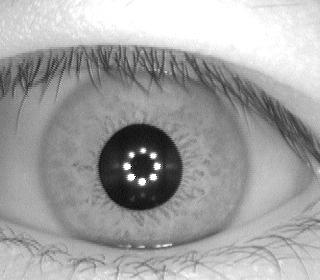

Supplement: Supplemental Information 1 [file peerj-cs-05-184-s001.zip › code9 PeerJ/Part1/gallery/g40.jpg]

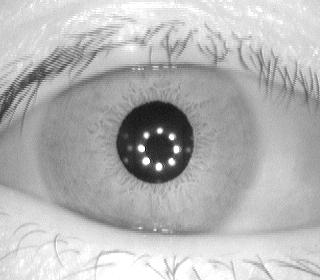

Supplement: Supplemental Information 1 [file peerj-cs-05-184-s001.zip › code9 PeerJ/Part1/gallery/g41.jpg]

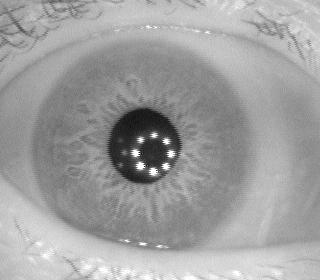

Supplement: Supplemental Information 1 [file peerj-cs-05-184-s001.zip › code9 PeerJ/Part1/gallery/g42.jpg]

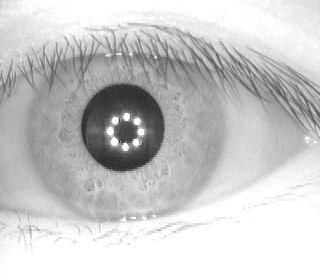

Supplement: Supplemental Information 1 [file peerj-cs-05-184-s001.zip › code9 PeerJ/Part1/gallery/g43.jpg]

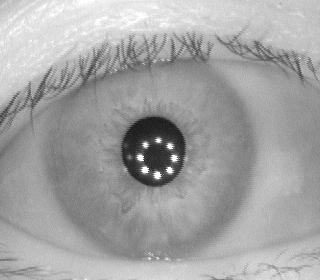

Supplement: Supplemental Information 1 [file peerj-cs-05-184-s001.zip › code9 PeerJ/Part1/gallery/g44.jpg]

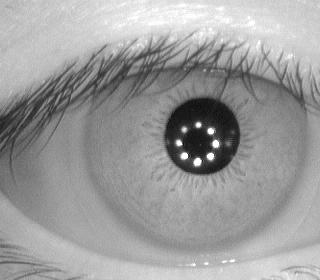

Supplement: Supplemental Information 1 [file peerj-cs-05-184-s001.zip › code9 PeerJ/Part1/gallery/g45.jpg]

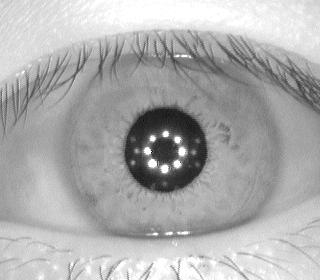

Supplement: Supplemental Information 1 [file peerj-cs-05-184-s001.zip › code9 PeerJ/Part1/gallery/g46.jpg]

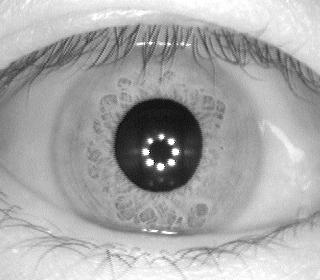

Supplement: Supplemental Information 1 [file peerj-cs-05-184-s001.zip › code9 PeerJ/Part1/gallery/g47.jpg]

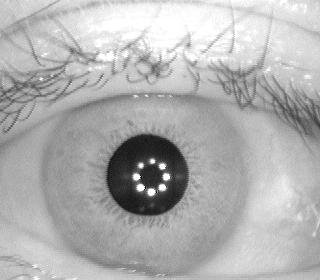

Supplement: Supplemental Information 1 [file peerj-cs-05-184-s001.zip › code9 PeerJ/Part1/gallery/g48.jpg]

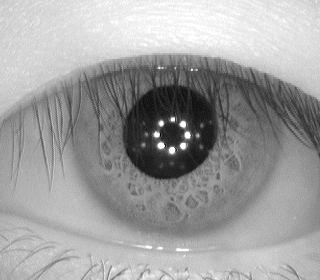

Supplement: Supplemental Information 1 [file peerj-cs-05-184-s001.zip › code9 PeerJ/Part1/gallery/g49.jpg]

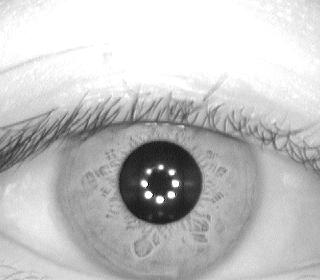

Supplement: Supplemental Information 1 [file peerj-cs-05-184-s001.zip › code9 PeerJ/Part1/gallery/g5.jpg]

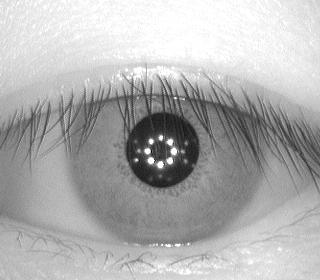

Supplement: Supplemental Information 1 [file peerj-cs-05-184-s001.zip › code9 PeerJ/Part1/gallery/g50.jpg]

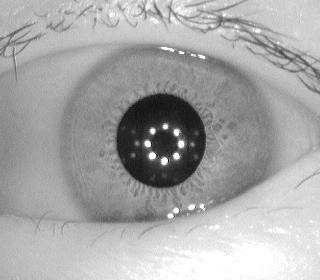

Supplement: Supplemental Information 1 [file peerj-cs-05-184-s001.zip › code9 PeerJ/Part1/gallery/g51.jpg]

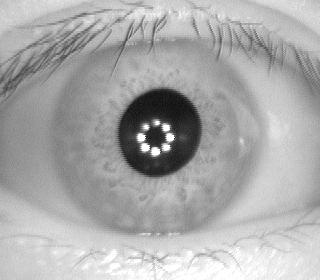

Supplement: Supplemental Information 1 [file peerj-cs-05-184-s001.zip › code9 PeerJ/Part1/gallery/g52.jpg]

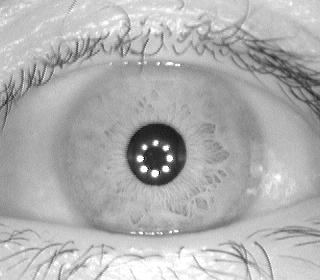

Supplement: Supplemental Information 1 [file peerj-cs-05-184-s001.zip › code9 PeerJ/Part1/gallery/g53.jpg]

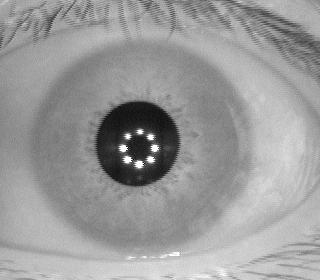

Supplement: Supplemental Information 1 [file peerj-cs-05-184-s001.zip › code9 PeerJ/Part1/gallery/g54.jpg]

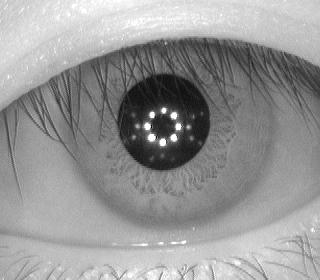

Supplement: Supplemental Information 1 [file peerj-cs-05-184-s001.zip › code9 PeerJ/Part1/gallery/g55.jpg]

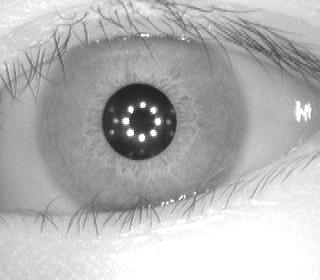

Supplement: Supplemental Information 1 [file peerj-cs-05-184-s001.zip › code9 PeerJ/Part1/gallery/g56.jpg]

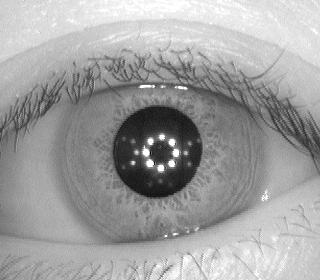

Supplement: Supplemental Information 1 [file peerj-cs-05-184-s001.zip › code9 PeerJ/Part1/gallery/g57.jpg]

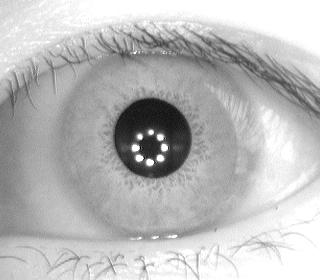

Supplement: Supplemental Information 1 [file peerj-cs-05-184-s001.zip › code9 PeerJ/Part1/gallery/g58.jpg]

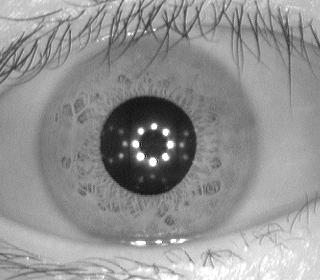

Supplement: Supplemental Information 1 [file peerj-cs-05-184-s001.zip › code9 PeerJ/Part1/gallery/g59.jpg]

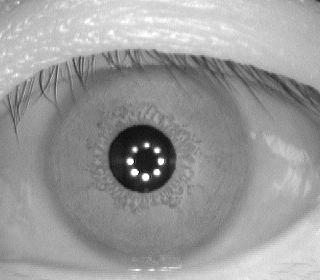

Supplement: Supplemental Information 1 [file peerj-cs-05-184-s001.zip › code9 PeerJ/Part1/gallery/g6.jpg]

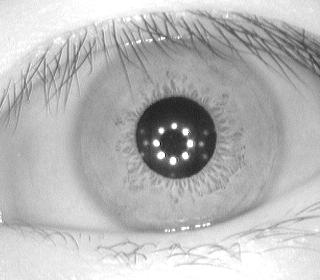

Supplement: Supplemental Information 1 [file peerj-cs-05-184-s001.zip › code9 PeerJ/Part1/gallery/g60.jpg]

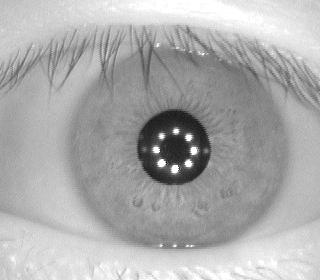

Supplement: Supplemental Information 1 [file peerj-cs-05-184-s001.zip › code9 PeerJ/Part1/gallery/g61.jpg]

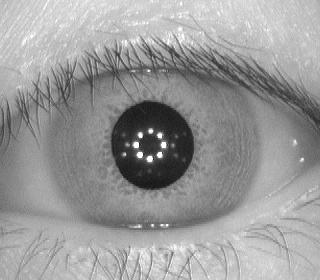

Supplement: Supplemental Information 1 [file peerj-cs-05-184-s001.zip › code9 PeerJ/Part1/gallery/g62.jpg]

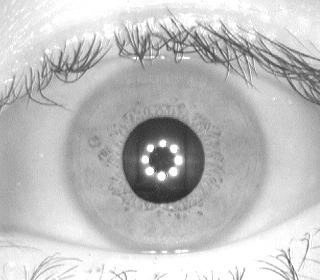

Supplement: Supplemental Information 1 [file peerj-cs-05-184-s001.zip › code9 PeerJ/Part1/gallery/g63.jpg]

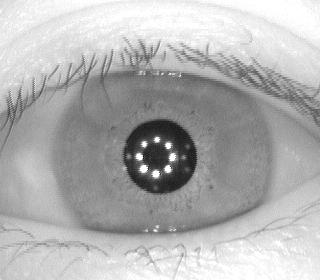

Supplement: Supplemental Information 1 [file peerj-cs-05-184-s001.zip › code9 PeerJ/Part1/gallery/g64.jpg]

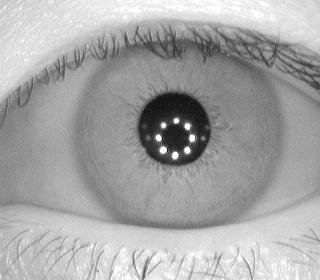

Supplement: Supplemental Information 1 [file peerj-cs-05-184-s001.zip › code9 PeerJ/Part1/gallery/g65.jpg]

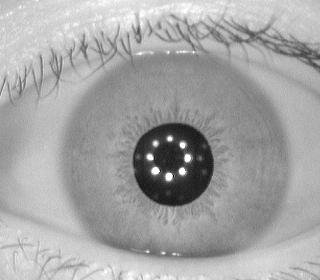

Supplement: Supplemental Information 1 [file peerj-cs-05-184-s001.zip › code9 PeerJ/Part1/gallery/g66.jpg]

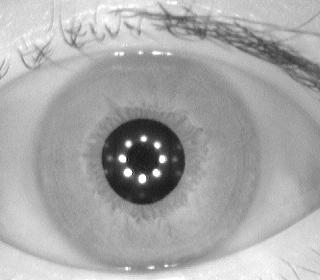

Supplement: Supplemental Information 1 [file peerj-cs-05-184-s001.zip › code9 PeerJ/Part1/gallery/g67.jpg]

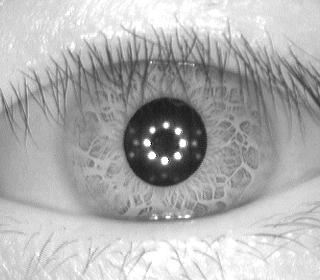

Supplement: Supplemental Information 1 [file peerj-cs-05-184-s001.zip › code9 PeerJ/Part1/gallery/g68.jpg]

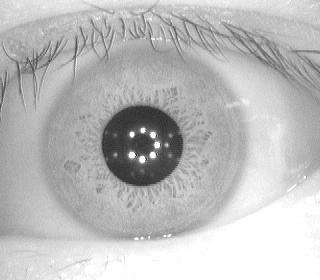

Supplement: Supplemental Information 1 [file peerj-cs-05-184-s001.zip › code9 PeerJ/Part1/gallery/g7.jpg]

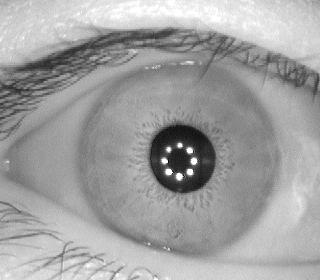

Supplement: Supplemental Information 1 [file peerj-cs-05-184-s001.zip › code9 PeerJ/Part1/gallery/g70.jpg]

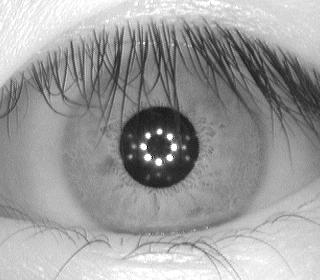

Supplement: Supplemental Information 1 [file peerj-cs-05-184-s001.zip › code9 PeerJ/Part1/gallery/g71.jpg]

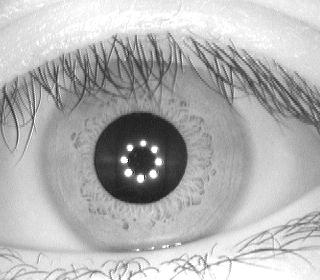

Supplement: Supplemental Information 1 [file peerj-cs-05-184-s001.zip › code9 PeerJ/Part1/gallery/g73.jpg]

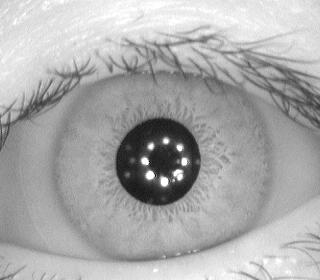

Supplement: Supplemental Information 1 [file peerj-cs-05-184-s001.zip › code9 PeerJ/Part1/gallery/g74.jpg]

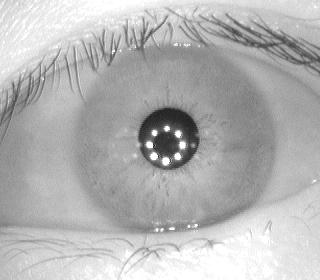

Supplement: Supplemental Information 1 [file peerj-cs-05-184-s001.zip › code9 PeerJ/Part1/gallery/g75.jpg]

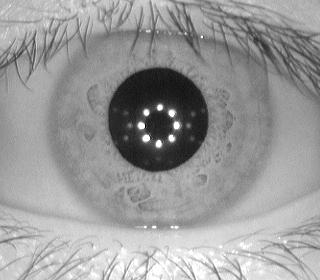

Supplement: Supplemental Information 1 [file peerj-cs-05-184-s001.zip › code9 PeerJ/Part1/gallery/g76.jpg]

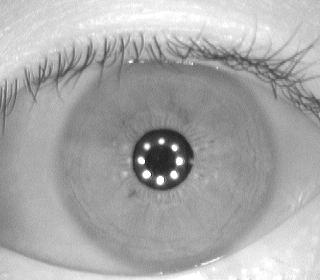

Supplement: Supplemental Information 1 [file peerj-cs-05-184-s001.zip › code9 PeerJ/Part1/gallery/g77.jpg]

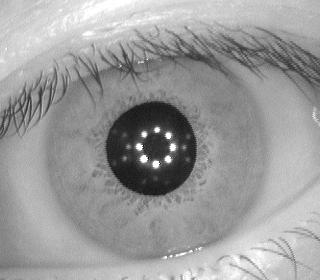

Supplement: Supplemental Information 1 [file peerj-cs-05-184-s001.zip › code9 PeerJ/Part1/gallery/g78.jpg]

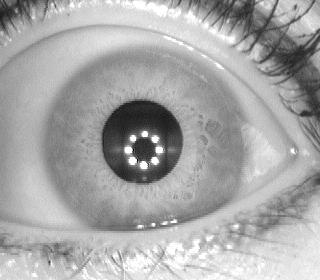

Supplement: Supplemental Information 1 [file peerj-cs-05-184-s001.zip › code9 PeerJ/Part1/gallery/g79.jpg]

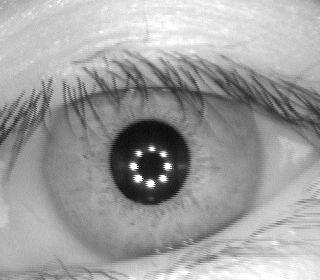

Supplement: Supplemental Information 1 [file peerj-cs-05-184-s001.zip › code9 PeerJ/Part1/gallery/g8.jpg]

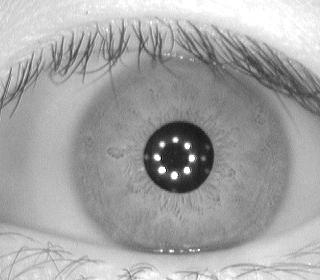

Supplement: Supplemental Information 1 [file peerj-cs-05-184-s001.zip › code9 PeerJ/Part1/gallery/g80.jpg]

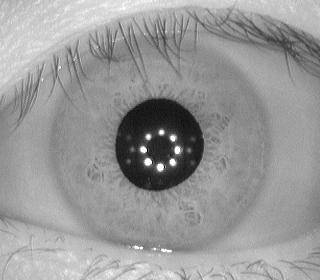

Supplement: Supplemental Information 1 [file peerj-cs-05-184-s001.zip › code9 PeerJ/Part1/gallery/g81.jpg]

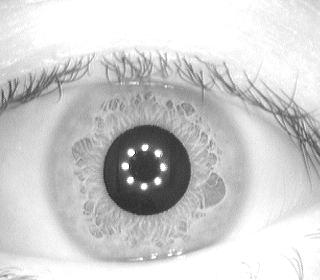

Supplement: Supplemental Information 1 [file peerj-cs-05-184-s001.zip › code9 PeerJ/Part1/gallery/g82.jpg]

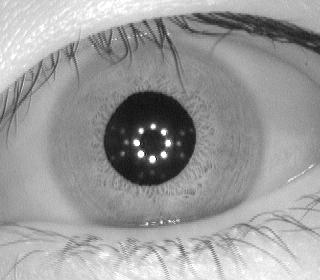

Supplement: Supplemental Information 1 [file peerj-cs-05-184-s001.zip › code9 PeerJ/Part1/gallery/g83.jpg]

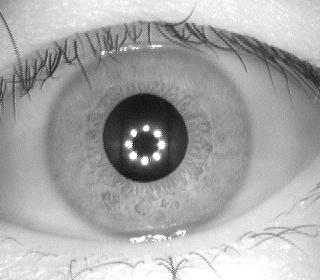

Supplement: Supplemental Information 1 [file peerj-cs-05-184-s001.zip › code9 PeerJ/Part1/gallery/g84.jpg]

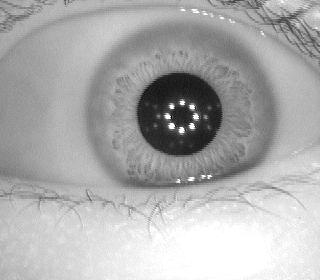

Supplement: Supplemental Information 1 [file peerj-cs-05-184-s001.zip › code9 PeerJ/Part1/gallery/g85.jpg]

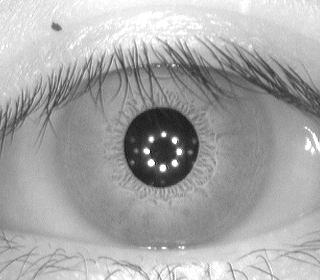

Supplement: Supplemental Information 1 [file peerj-cs-05-184-s001.zip › code9 PeerJ/Part1/gallery/g86.jpg]

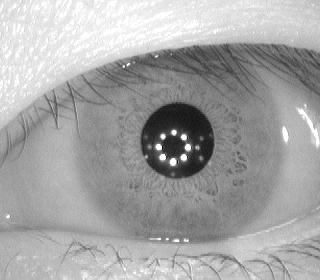

Supplement: Supplemental Information 1 [file peerj-cs-05-184-s001.zip › code9 PeerJ/Part1/gallery/g87.jpg]

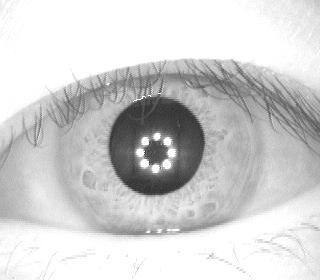

Supplement: Supplemental Information 1 [file peerj-cs-05-184-s001.zip › code9 PeerJ/Part1/gallery/g88.jpg]

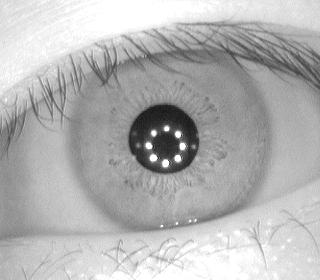

Supplement: Supplemental Information 1 [file peerj-cs-05-184-s001.zip › code9 PeerJ/Part1/gallery/g89.jpg]

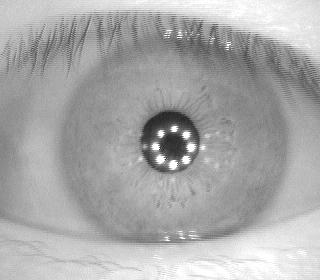

Supplement: Supplemental Information 1 [file peerj-cs-05-184-s001.zip › code9 PeerJ/Part1/gallery/g9.jpg]

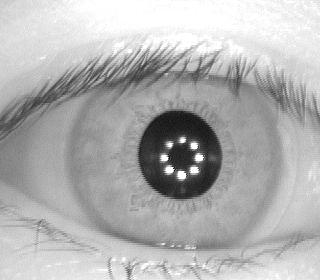

Supplement: Supplemental Information 1 [file peerj-cs-05-184-s001.zip › code9 PeerJ/Part1/gallery/g90.jpg]

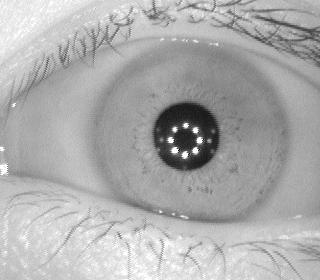

Supplement: Supplemental Information 1 [file peerj-cs-05-184-s001.zip › code9 PeerJ/Part1/gallery/g91.jpg]

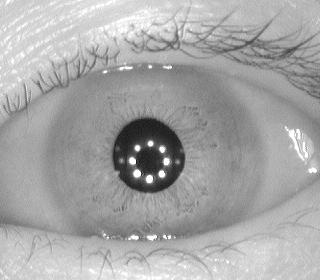

Supplement: Supplemental Information 1 [file peerj-cs-05-184-s001.zip › code9 PeerJ/Part1/gallery/g92.jpg]

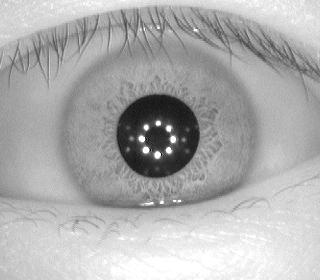

Supplement: Supplemental Information 1 [file peerj-cs-05-184-s001.zip › code9 PeerJ/Part1/gallery/g93.jpg]

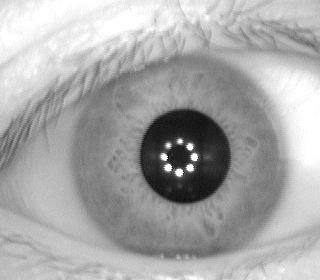

Supplement: Supplemental Information 1 [file peerj-cs-05-184-s001.zip › code9 PeerJ/Part1/gallery/g94.jpg]
